# A novel feedback loop: CELF1/circ-CELF1/BRPF3/KAT7 in cardiac fibrosis

**DOI:** 10.1016/j.apsb.2025.07.036

**Published:** 2025-07-29

**Authors:** Yuan Jiang, Bowen Zhang, Bo Zhang, Xinhua Song, Xiangyu Wang, Wei Zeng, Liyang Zuo, Xinqi Liu, Zheng Dong, Wenzheng Cheng, Yang Qiao, Saidi Jin, Dongni Ji, Xiaofei Guo, Rong Zhang, Xieyang Gong, Lihua Sun, Lina Xuan, Berezhnova Tatjana Alexandrovna, Xiaoxiang Guan, Mingyu Zhang, Baofeng Yang, Chaoqian Xu

**Affiliations:** a State Key Laboratory of Frigid Zone Cardiovascular Diseases (SKLFZCD), Department of Pharmacology (State Key Labratoray-Province Key Laboratories of Biomedicine-Pharmaceutics of China, Key Laboratory of Cardiovascular Research, Ministry of Education), College of Pharmacy, Harbin Medical University, Harbin 150081, China; b Department of Pharmacology, State-Province Key Laboratories of Biomedicine Pharmaceutics of China, Key Laboratory of Cardiovascular Medicine Research, Ministry of Education, College of Pharmacy, Harbin Medical University, Harbin 150081, China; c Department of Biopharmaceutical Sciences, College of Pharmacy, Harbin Medical University, Harbin 150081, China; d Institute of Clinical Pharmacy, the Second Affiliated Hospital, Harbin Medical University, Harbin 150081, China; e Department of Ultrasound, the Second Affiliated Hospital of Harbin Medical University, Harbin 150081, China; f Department of Pharmacology of Voronezh State Medical University, Voronezh 394018, Russia; g Institute of Clinical Pharmacy, the First Affiliated Hospital of Harbin Medical University, Harbin 150001, China

**Keywords:** CELF1, Circ-CELF1, Cardiac fibrosis, BRPF3, KAT7, Feedback loop, Histone acetylation, Therapeutic target

## Abstract

Cardiac fibrosis is characterized by an elevated amount of extracellular matrix (ECM) within the heart. However, the persistence of cardiac fibrosis ultimately diminishes contractility and precipitates cardiac dysfunction. Circular RNAs (circRNAs) are emerging as important regulators of cardiac fibrosis. Here, we elucidate the functional role of a specific circular RNA CELF1 in cardiac fibrosis and delineate a novel feedback loop mechanism. Functionally, circ-CELF1 was involved in enhancing fibrosis-related markers' expression and promoting the proliferation of cardiac fibroblasts (CFs), thereby exacerbating cardiac fibrosis. Mechanistically, circ-CELF1 reduced the ubiquitination-degradation rate of BRPF3, leading to an elevation of BRPF3 protein levels. Additionally, BRPF3 acted as a modular scaffold for the recruitment of histone acetyltransferase KAT7 to facilitate the induction of H3K14 acetylation within the promoters of the *Celf1* gene. Thus, the transcription of *Celf1* was dramatically activated, thereby inhibiting the subsequent response of their downstream target gene *Smad7* expression to promote cardiac fibrosis. Moreover, *Celf1* further promoted *Celf1* pre-mRNA transcription and back-splicing, thereby establishing a feedback loop for circ-CELF1 production. Consequently, a novel feedback loop involving CELF1/circ-CELF1/BRPF3/KAT7 was established, suggesting that circ-CELF1 may serve as a potential novel therapeutic target for cardiac fibrosis.

## Graphical abstract

A novel feedback loop involving CELF1/circ-CELF1/BRPF3/KAT7 was studied in cardiac fibrosis pathogenesis.

**Figure:**
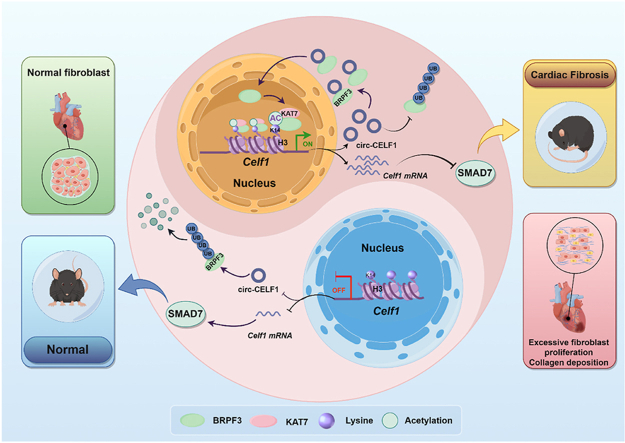
A novel feedback loop involving CELF1/circ-CELF1/BRPF3/KAT7 in cardiac fibrosis pathogenesis.

## 1 Introduction

Cardiac fibrosis plays a crucial role in the advancement of cardiovascular disorders, including myocardial infarction (MI), cardiac hypertrophy, doxorubicin-induced cardiomyopathy, diabetic cardiomyopathy as well as diastolic heart failure^1^^,^^2^. Ultimately, this leads to excessive deposition of advanced cardiac extracellular matrix (ECM) and results in heart failure^3^. The primary source of collagen fibers in cardiac fibrosis is cardiac fibroblasts (CFs). The activated fibroblasts differentiate into myofibroblasts and secrete contractile proteins as well as ECM-degrading metalloproteinases (MMPs), ultimately leading to the development of cardiac fibrosis^4^. Several growth factors and cytokines, such as transforming growth factor-*β* (TGF-*β*), angiotensin II, and bone morphogenetic protein, serve as critical driving forces culminating in the cardiac fibrotic response^5^^,^^6^. More recently, the exploration of novel molecular targets^7^, epigenetic alterations^8^, as well as regulatory RNAs^9^ have unveiled intriguing strategies for tackling the development of cardiac fibrosis^10^.

Circular RNAs (circRNAs) are generated through the back splicing of protein-coding gene exons or introns from protein-coding genes, characterized by their distinctive shape lacking a 5′ cap or 3′ poly A tail^11^. Functionally, circRNAs participate in intracellular signal transduction by interacting with DNA, sponging miRNAs, modulating protein translation, and encoding small peptides^12^, ^13^, ^14^, ^15^, ^16^. Emerging studies have proposed that circRNAs can interact with host genes, thereby participating in a feedback loop pathway within the mechanism of genetic transcription^17^. CircRNAs, in this way, participate in the pathogenesis of various cancer-related diseases^18^, ^19^, ^20^, however, the mechanisms in cardiac fibrosis remain to be further elucidated.

Gene expression and transcriptional regulation are modulated by histone modifications, including histone acetylation and methylation, as well as DNA methylation^21^^,^^22^. These epigenetic events may be involved in the complex regulatory interaction between host genes and circRNA expression. Histone acetyltransferases, also known as HATs, are enzymes that catalyze the substitution of an acetyl group from acetyl-CoA to lysine residues of histones^23^. These histones can be found in a wide variety of biological situations and have an essential function in regulating the sequence of transcriptional events^24^. The BRPF family, comprising BRPF1, BRPF2, and BRPF3, function as scaffold proteins for the HATs complex, thereby improving its acetyltransferase activity and transcriptional activation^25^. Among the BRPF family, BRPF3 plays pivotal roles in the regulation of gene expression, including DNA replication, gene transcription, nucleosome assembly, and maintenance of pluripotency in embryonic stem cells^26^. Nevertheless, whether BRPF3 affects the pathological process of cardiac fibrosis remains unknown.

Within the scope of this investigation, we have successfully identified a unique circular RNA (circRNA) named circ-CELF1, which was upregulated in MI mouse heart tissues and in CFs treated with TGF-*β*1. Furthermore, we found that circ-CELF1 exhibited profibrotic properties both *in vitro* and *in vivo*. Mechanistically, we revealed that circ-CELF1 enhanced BRPF3 expression and improved the stability of BRPF3 protein by attenuating its ubiquitination-mediated degradation. Interestingly, BRPF3 was found to translocate into the cell nucleus and recruited histone acetyltransferase KAT7, leading to an elevation in H3K14 acetylation levels within *Celf1* promoter regions. Therefore, the transcription of *Celf1* was significantly activated. On the one hand, CELF1 inhibited the subsequent response of their downstream target gene *Smad7* expression to promote cardiac fibrosis. On the other hand, *Celf1* further facilitated *Celf1* pre-mRNA transcription and back-splicing, thus establishing a feedback loop for circ-CELF1 production. Collectively, the novel circ-CELF1, which regulates histone modification to activate *Celf1*, may serve as a potential novel therapeutic target for mitigating cardiac fibrosis.

## 2 Materials and methods

### 2.1 Experimental models

Male C57BL/6J mice (20–25 g) were procured from Liaoning Changsheng Biotechnology Co., Ltd. The rodents were kept in a rodent room and provided with nourishment and water for a week before the experiments. All experimental processes were carried out in line with the Institutional Animal Care and Use Committee (IACUC) of Harbin Medical University (approval No. IRB5017622).

### 2.2 MI model development and adeno-associated virus (AAV) construction

Adult C57BL/6J mice were anesthetized with avertin (2% tribromoethanol, 10 μL/g) administered *via* intraperitoneal injection. Anesthesia adequacy was verified by no tail pinch response. After intubation, the cannula was attached to a volume-cycled rodent ventilator (Ugo Basile SRL 3), ventilated at a rate of 110 breaths/min. In the MI model, the left anterior descending coronary artery was ligated 1–2 mm from the lower margin of the left atrial appendage, ligated with a 7-0 nylon suture (Shanghai Medical Suture, Shanghai, China). Similar surgical procedures were performed on Sham-operated mice without the LAD ligation. The experiments were conducted by a single researcher to guarantee the consistency as well as stability of the experimental procedure, as well as to maintain consistent levels of MI among the different groups.

The AAV9 system, utilizing the periostin core promoter, was employed to facilitate circ-CELF1 upregulation and silencing in mouse CFs. The AAV9 containing a short interfering RNA directed against circ-CELF1 (AAV9-sh-circ-CELF1) for loss of function, a scrambled fragment as a negative control (AAV9-sh-NC), and the AAV9 with the complete sequence of circ-CELF1 (AAV9-circ-CELF1) for gain of function, along with an empty vector (AAV9-Vector) as a negative control, were all synthesized by GENECHEM (Shanghai, China). AAV9-sh-circ-CELF1 or AAV9-sh-NC, containing short hairpin RNA or scrambled sequences targeting circ-CELF1, was administered *via* the tail vein of animal models to create a circ-CELF1 knockdown model over three weeks. Subsequently, the mice were subjected to MI for four weeks. The mice were administered tail vein injections of either AAV9-circ-CELF1 or AAV9-Vector to induce circ-CELF1 upregulation for 7 weeks. The titer of the AAV9 was 1.2 × 10^11^ U/mL.

### 2.3 Echocardiography

Mice were anesthetized with avertin (2%, 10 μL/g, intraperitoneal injection) and subsequently positioned on a platform to maintain their heart rate within the range of 450–550 beats/min. In order to evaluate the heart's structure, transthoracic echocardiography was employed, with the M-mode cursor set at the maximum LV area to measure ejection fraction (EF), fractional shortening (FS), LV end-systolic diameter (LVID;s) and LV end-diastolic diameter (LVID;d).

### 2.4 Masson's trichrome staining and histological staining

The hearts of mice with MI and sham mice were isolated and fixed in 4% paraformaldehyde (HX-6151, Biosharp, Fujian, China) for 24 h at room temperature, followed by performing Masson's trichrome staining according to the manufacturer's instructions (G1340-7 × 100 mL, Solarbio, Beijing, China). The percentage of the fibrotic area was inspected by using a light microscope (Olympus) with blue staining to indicate the disposition of collagen.

Before being embedded in paraffin, cardiac tissue specimens were fixed for a whole night in a 4% paraformaldehyde solution, and the paraffin slices were acquired with a 5 μm thickness. Following this, the sections were dewaxed using xylene and underwent a series of ethanol washes at different concentrations. Immunohistochemical staining was carried out using antibodies targeting *α*-SMA (AF1032, Affinity Biosciences, USA). Image acquisition was performed using a light microscope (Olympus).

### 2.5 CFs culture

Primary CFs were extracted from mice that were between one and three days old and purchased from the Animal Experimentation Centre of the Second Affiliated Hospital of Harbin Medical University in Harbin, China. The hearts were initially rinsed with PBS physiological buffer solution containing 1% penicillin–streptomycin and then cut into uniform chunks. The samples were subsequently subjected to overnight digestion using a trypsin-EDTA solution (Seven-SC109-01, Seven, Beijing, China). The subsequent step involved a re-digestion process utilizing preheated collagenase type II (BIS-BS164-100 mg, Biosharp, Fujian, China) at 37 °C. Centrifugal force was used to recover the cells, and then they were resuspended in DMEM (PNS-PM150210, Pricella, Wuhan, China) that had been treated with 10% fetal bovine serum (FBS, Biological Industries, Israel) and 1% penicillin/streptomycin. After incubating for 1–1.5 h, non-adherent cells and weakly adherent cardiomyocytes were eliminated by differential speed centrifugation, resulting in the isolation of CFs. For 24 h, the cells were grown in DMEM supplemented with 10% FBS, 100 U/mL of penicillin, as well as 100 μg/mL of streptomycin.

### 2.6 RNA isolation and qPCR

Total RNA was extracted from cultured CFs and cardiac tissue using TRIZOL reagent (IN-15596-026, Thermo Fisher, Waltham, MA, USA). After following the manufacturer's procedure, a PARISTM kit (Thermo Fisher Scientific, CA, USA) was used to isolate cytoplasmic and nuclear RNAs. A total of 500 ng of total RNA was reverse transcribed into cDNA using TransScript Uni All-in-One First-Strand cDNA Synthesis SuperMix (MN-MR05101M, Toyobo, Osaka, Japan). The levels of circ-CELF1, *Celf1*, *Fn1*, Collagen1, *Brpf3*, *Kat7*, and *β*-actin were measured by qPCR using SYBR Green I (B5329550005, Sangon Biotech, Shanghai, China) on the ABI 7500 Fast system. For mRNA quantification, *β*-actin was used as an internal control. The relative mRNA levels were computed using the Ct values, which were obtained by determining the threshold cycle (Ct). The 2^–ΔΔCT^ technique was used to compute the data. Correlations were analyzed with Pearson's test, and *P* values < 0.05 were considered significant. The primers utilized in this study are listed in Supporting Information Table S1.

### 2.7 Western blot (WB) analysis

Through the utilization of RIPA buffer (MA0151-100 mL, meilunbio, Dalian, China), the entire protein was recovered from CFs as well as heart tissues. After transferring samples onto nitrocellulose filter membranes, they were treated with 10% SDS-PAGE. After blocking the nitrocellulose filter membranes with 5% skim milk at a temperature of 25 °C for 1.5 h. After that, the membranes were subjected to an incubation with the primary antibodies. Following a total of four washes with PBST, we incubated the membranes for 1 h with polyclonal secondary antibodies 800M (926-32210-100 μL, Licor, Nebraska, USA) or 800R (926-32211-100 μL, Licor, Nebraska, USA). These antibodies were either mouse or rabbit-specific. Protein band gray levels were examined using the ODYSSEY equipment (LI-COR, DLx, USA). Supporting Information Table S2 details the antibodies utilized in this investigation.

### 2.8 Small interfering RNA (siRNA)

SiRNAs targeting circ-CELF1, *Brpf3*, *Kat7*, *and Celf1*, along with a scrambled control, were synthesized by RIBOBIO (Guangzhou, China). The cells were seeded in 6-well plates and incubated for 24 h. Subsequently, cells were transfected with siRNAs by using Lipo2000 (11668-019, Thermo Fisher, Waltham, MA, USA) at a final concentration of 100 nmol/L. The CFs with a confluence ranging from 70% to 80% were transfected using a mixture of 10 μL of siRNA and Lipofectamine 2000® siRNA at a ratio of 1:1. After each reagent was incubated for 5 min on its own, they were mixed and left to incubate at room temperature for another 15 min. After that, the cells were treated with the siRNA-transfection reagent mixture and left to incubate for 48 h before the next set of experiments. The siRNA sequences are provided in Supporting Information Table S3.

### 2.9 Circ-CELF1 overexpression in CFs

The circ-CELF1 overexpression vector was created by GENECHEM (Shanghai, China) following standard procedures. A 605 bp fragment containing the gene sequence of circ-CELF1 and the upstream and downstream intron cyclization components was obtained by performing PCR with the following primers:

5′-CTCCCCACCATCACTTTTTAGCTCAAAGAAAATGAACGGCAC-3′ and 5′- AACTTGGGAAATTCTTTTGTACTGCTAAGTACTGGGGTCCAAGTG-3′.

The circ-CELF1 overexpressing plasmid is constructed using the GV728 vector and digested with AgeI/BamHI restriction enzymes. It contains the following elements: CMV enhancer-left circular frame-MCS-right circular frame-SV40-puromycin.

### 2.10 FISH mapping of circ-CELF1 in CFs

The intracellular distribution of circ-CELF1 in CFs was determined using fluorescence *in situ* hybridization (FISH) assay. Cy3-labeled circ-CELF1 probes were conducted by GenePharma (Shanghai, China). The signals from the probes were detected using a FISH Kit (RIBOBIO Guangzhou, China) following the manufacturer's instructions. Briefly, the CFs were fixed in 4% paraformaldehyde and subsequently incubated overnight at 37 °C with Cy3-labeled circ-CELF1 probes. The CFs were subsequently rinsed with Saline Sodium Citrate Buffer at a temperature of 42 °C on the following day. The nucleus was then subjected to DAPI staining (SLB-C0065-10 mL, Solarbio, Beijing, China) for 15 min. The U6 FISH Probe was utilized for nuclear localization, the 18S FISH Probe was employed to determine cytoplasmic localization, the NC-probe served as a control, and DAPI (4,6-diamidino-2-phenylindole) was applied for nuclear labeling.

Primer and probe sequences were as follows:

mmu-circ-CELF1: CGTTCATTTTCTTTGAGTGCTAAGTACTGGGG.

Colocalization was analyzed using the ScatterJ plug-in in ImageJ, with the scatter plot axes representing the gray values of each pixel in the respective channels. Pearson's coefficient ranges from −1 to 1, encompassing a comprehensive spectrum of values. The proximity of the results to 1 indicates a stronger correlation.

### 2.11 Immunofluorescence

After four washes with cold PBS, the CFs were cultured on sterilized glass coverslips in 24-well plates. For 1 h, the CFs were immersed in 4% paraformaldehyde to fix them. Cells were permeabilized with 0.3% Triton X-100 for 1 h, followed by incubation with normal goat serum at 37 °C for 1 h to block the membrane. CFs were incubated overnight at 4 °C with primary antibodies, then incubated for 2 h at 37 °C with DyLight 594 goat anti-mouse(ab150116, abcam, Cambridgeshire, UK), DyLight 594 goat anti-rabbit (ab150080, abcam, Cambridgeshire, UK), and DyLight 488 goat anti-rabbit (ab150077, abcam, Cambridgeshire, UK) secondary antibodies.

Nuclei were DAPI-stained for 15 min at room temperature (SLB-C0065-10 mL, Solarbio, Beijing, China). Antibody details are provided in Table S2.

The mean gray value was detected using ImageJ, where the mean gray value (Mean) is calculated as the Integrated Density (IntDen) divided by the Area. The stained sections were analyzed in various regions, with 9 to 39 images and measurements of 10 to 20 individual cells per region. The default threshold was employed for background correction to mitigate the error introduced by manual threshold selection.

### 2.12 EdU fluorescence staining

To detect cardiac fibroblast proliferation, the Cell-Light EdU Kit (KTA2031, abbkine, CA, USA) was employed in accordance with the manufacturer's instructions. EdU was added to the plate and incubated for an additional 12 h to measure the newly synthesized DNA. Thereafter, the nuclei were incubated at room temperature for 15 min with DAPI (SLB-C0065-10 mL, Solarbio, Beijing, China). Fluorescence microscopy (Zeiss, U-RFL-T, Jena, Germany) was used to analyze the alterations in proliferation. The blue dots show the existence of the nucleus, while the red dots represent freshly created DNA.

### 2.13 Co-immunoprecipitation

CFs were lysed in Co-IP lysis buffer supplemented with a protease inhibitor. The protein complexes were captured using 50 μL of Protein A/G Magnetic Beads (HY-K0202-1 mL, MCE, Shanghai, China) in conjunction with the flag antibody (MedChemExpress, China) at 4 °C overnight. The beads were then subjected to washing, followed by elution and subsequent collection of immunoprecipitates and whole-cell lysates. Before immunoblotting with the appropriate antibodies, the protein samples were electrophoresed on a 10% SDS-PAGE gel. A negative control was established using IgG.

### 2.14 Pull-down assay

RNA pull-down assays were performed with the BersinBioTM RNA Pull-down Kit (catalog no. Bes5102, BersinBio, Guangzhou, China). Briefly, the biotinylated circ-CELF1 and NC probe were subjected to denaturation in a 90 °C water bath for 2 min. Circ-CELF1 and NC probes were mixed with streptavidin beads and added to the cell sample. The resultant mixture was thereafter incubated at ambient temperature for 2 h. Refrain from the mixture for 1 min to capture magnetic beads and discard the supernatant. After being washed, the sample was incubated at a temperature of 37 °C for 2 h. The protein samples were utilized for WB analysis.

The following describes the sequences of the probes:

NC: UUGUACUACACAAAAGUACUG

mmu-circ-CELF1: TTTGAGTGCTAAGTACTGGGGTCCAAGTGTG.

### 2.15 RIP assay

RIP assay was performed with the RNA immunoprecipitation Kit (catalog no. Bes5101, BersinBio, Guangzhou, China). The 2 × 10^7^ CFs were washed with 4 mL of PBS, and the supernatant was centrifuged at room temperature for 7 min at a speed of 3000 rpm (Desktop centrifuge, Hitachi-CT15RE, Japan) to collect the cells. After that, the CFs were broken down using RIP buffer and left to incubate at 4 °C overnight with antibodies conjugated to protein A/G beads. The antibodies used were anti-BRPF3 (5 μg; Thermo Fisher Scientific, USA) and IgG (5 μg; catalog no. Bes5101, BersinBio, Guangzhou, China). A total of 1000 ng of extracted RNA, IP, and input samples were then transformed into cDNA. We used the ABI 7500 fast real-time PCR instrument to quantify the circ-CELF1 levels.

### 2.16 CHIP assay

Chromatin Immunoprecipitation assays were performed with the CHIP assay kit (catalog no. P2078, Beyotime Biotechnology, Shanghai, China). Briefly, CFs were subjected to cross-linking using a 37% formaldehyde solution for 10 min at 37 °C, followed by quenching with 125 mmol/L glycine. The cells were centrifuged at 4 °C for 800 × *g*–1000 × *g* for 2 min to achieve complete cell precipitation. Subsequently, incubate the samples in an ice bath for 10 min to ensure complete cell lysis. The DNA fragments ranging from 400 to 800 bp were obtained through sonication and incubation at 65 °C for 4 h to disrupt the protein-DNA cross-linking. After centrifugation at 12,000 × *g* for 10 min at 4 °C, the lysate was subsequently subjected to immunoprecipitation using anti-H3K14ac and IgG antibodies, followed by overnight incubation at 4 °C. Protein A/G beads (60 μL) were added to the antibody-lysate mixture and incubated for an additional hour at 4 °C. The beads were washed, and DNA fragments were eluted, purified using the DNA Purification Kit (D0033; Beyotime Biotechnology, Shanghai, China), and analyzed by qPCR.

### 2.17 4D-FastDIA quantitative proteomics

The 4D-FastDIA quantitative proteomics analysis experiments were conducted at Jingjie PTM BioLab (Hangzhou) Co., Inc. In brief, (1) Construct a sample-specific protein database according to the origin of the samples, and subsequently perform database searches utilizing analytical software. (2) Based on the outcomes of the database search, perform quality control analysis at both the peptide and protein levels. (3) Conduct a comprehensive quantitative analysis of proteins, including the evaluation of quantitative distribution and repeatability. Additionally, present the distribution results of sample quantitative intensity values clearly and systematically. The normalized intensity (I) of the protein in different samples was transformed by centralization to obtain the relative quantitative value (R) of the protein in different samples. The calculation formula is as follows: The calculation formula is as follows: *Rij* = *Iij*/Mean (*Ij*). (4) where i represents the sample and j represents the protein. Functional annotations were conducted on the identified proteins using a standardized and comprehensive approach.

### 2.18 Nucleocytoplasmic separation assay proteins

The Nuclear and Cytoplasmic Protein Extraction Kit (P0027, Beyotime Biotechnology, Shanghai, China) was utilized to perform cytoplasmic and nuclear protein separation in accordance with the provided instructions. Under low osmotic pressure circumstances, the CFs are completely enlarged using cytoplasmic protein extraction reagents A as well as B. After that, cytoplasmic proteins are released by disrupting the cell membrane, and the nucleus is precipitated by centrifugation. The final step in nuclear protein extraction was the use of a high-salt reagent developed for this purpose.

### 2.19 Wound healing assay

For wound healing assay, cells were grown in 6-well plates and transfected with circ-CELF1 siRNA, circ-CELF1 overexpression plasmid and BRPF3 siRNA as previously described. A standardized wound was generated using a 10 μL pipette tip. Pictures were taken under the microscope at 0 and 48 h.

### 2.20 RNA stability assays

CFs were cultured in 12-well plates and subsequently transfected with CELF1 siRNA according to the protocol described above. After 24 h of transfection, the CFs were treated with actinomycin D (5 mg/mL, catalog no. HY-17559, Sigma–Aldrich, Burlington, USA) for 2, 4, 6 and 8 h before collection. Total RNA was extracted with TRIzol and analyzed by qPCR.

### 2.21 Lactate dehydrogenase release assay

Cardiomyocytes were cultured in 96-well plates and subsequently incubated in conditioned medium derived from CFs overexpressing circ-CELF1. The LDH assay kit (C0016, Beyotime Biotechnology, Shanghai, China) was used in the following experiments. Prior to the lactate dehydrogenase (LDH) experiment, 20 μL of LDH release agent was added to the control group and incubated for 1 h. A total of 120 μL of cell supernatant was collected and centrifuged at 400 × *g* for 5 min. Then, 60 μL of the LDH assay working solution was added to the samples, followed by incubation for 30 min. Finally, the absorbance was measured at 490 nm using a spectrophotometric microplate reader.

### 2.22 CCK8 assays

Cell viability was assessed using a Cell Counting Kit-8 (BS350A, Biosharp, Anhui, China) according to the manufacturer's instructions. Following the treatment, CCK8 working solution (comprising 90% DMEM and 10% CCK8) was added to the cardiomyocytes, which were then incubated for 2 h. The absorbance at 450 nm was measured using a microplate reader (Tecan, Switzerland) to determine the cell viability.

### 2.23 Surface plasmon resonance (SPR) analysis

The equilibrium-binding constant of KAT7 and BRPF3 was determined by SPR analysis performed on a BIAcore 8K instrument. In brief, the 50 μg ligand protein-KAT7 (purchased from MedChemExpress, Shanghai, China) was dissolved in sodium acetate buffer to achieve a concentration of 50 μg/mL. Subsequently, the protein was immobilized on the chip surface *via* a flow rate of 10 μL/min to construct the coupling map. The 50 μg BRPF3 protein (purchased from MedChemExpress, Shanghai, China) was serially diluted in a 96-well plate and subsequently coupled with the target protein on the chip, ranging from the lowest to the highest concentration. The flow rate was maintained at 30 μL/min for 150 s. Following each concentration measurement, the chip was regenerated using a 10 mmol/L glycine hydrochloride solution (pH 2.0) for 5 min. This regeneration process was repeated until all corresponding analyte concentrations had been successfully analyzed. The data were analyzed using the Biacore Insight evaluation software (Cytiva, Marlborough, MA, USA) to determine binding and dissociation constants.

### 2.24 Data analysis

Data are expressed as mean ± standard deviation (SD). The normality of expression values was assessed before statistical analysis. The Student's *t*-test was used for two-group comparisons, while One-way ANOVA with the Bonferroni *post hoc* test was applied for multiple groups. Nonparametric tests (Mann–Whitney U or Kruskal–Wallis with Dunn post-test) were used for non-normally distributed data (*P* < 0.05). Analysis was done with GraphPad Prism 8.0.

## 3 Results

### 3.1 Identification and characterization of the conserved circular RNA CELF1 in cardiac fibrosis

**Figure 1:**
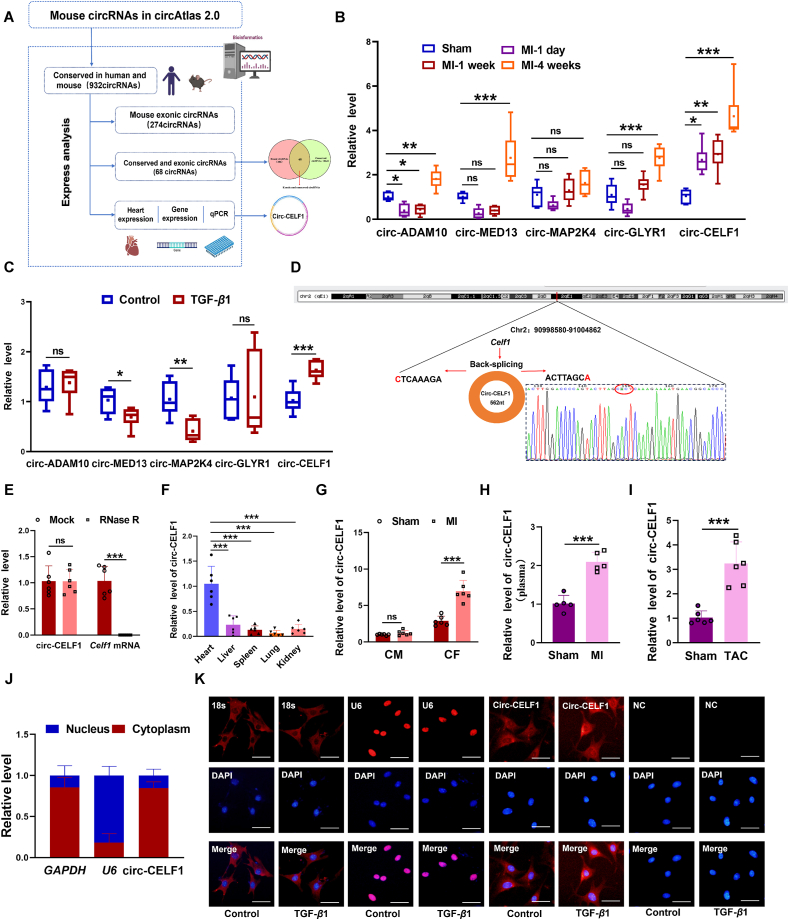
Circ-CELF1 is identified and characterized in the cardiac fibrosis model. (A) The screening procedure of circ-CELF1 was conducted *via* the database. (B) The levels of circRNAs were quantified using qPCR at 1 day, 1 week, and 4 weeks post-MI (*n* = 6). (C) The levels of five selected circRNAs in CFs treated with TGF-*β*1 (*n* = 6). (D) Schematic diagram illustrating the genomics information of circ-CELF1, which originates from the splicing of *Celf1* gene located at Chr2:90998580-91004862. (E) qPCR analysis of the levels of circ-CELF1 and linear *Celf1* mRNA in CFs following treatment with RNase R (*n* = 6). (F) qPCR analysis showing relative levels of circ-CELF1 in different tissues of mice (*n* = 6). (G) qPCR assays showing circ-CELF1 expression in the CFs and CMs (*n* = 6). (H) qPCR analysis of circ-CELF1 expression in mouse plasma (*n* = 5). (I) The expression level of circ-CELF1 was quantified by qPCR six weeks after transverse aortic constriction (TAC) (*n* = 6). (J) Cytoplasmic/Nuclear fractionation followed by qPCR indicates the subcellular localization of circ-CELF1 in CFs (*n* = 5). (K) FISH analysis reveals the cytoplasmic localization of circ-CELF1 in CFs. Circ-CELF1 probes (Cy3, red) and nuclei (DAPI, blue) were stained, 18s was used as a cytoplasmic marker, U6 served as a marker for the nucleus, and NC was included as a negative control. Each data point in the figure represents a unique biological replicate. The data are presented as the mean ± SD. Statistical analysis was performed with one-way ANOVA followed by Bonferroni correction and Student's *t* test for two means. ∗*P* < 0.05, ∗∗*P* < 0.01, ∗∗∗*P* < 0.001.

To investigate the role of circRNAs in the development of cardiac fibrosis, the circAtlas database was reanalyzed. The circRNAs expression data obtained from the circAtlas dataset underwent subsequent analysis and experimentation as described below. (1) Highly conserved circRNAs between mice and humans (932circRNAs). (2) CircRNAs derived from exonic regions (274 circRNAs). (3) Highly conserved and exonic circRNAs (68 circRNAs). (4) Most abundant in mouse heart and high host genes expression (5 circRNAs). From this stepwise screening, we prioritized five circRNAs for analysis: circ-ADAM10, circ-MED13, circ-MAP2K4, circ-GLYR1, and circ-CELF1 (Fig. 1A). Subsequently, we established MI models at various time points and conducted echocardiography analysis, which revealed that the EF and FS in mice with MI at 1-day, 1-week, and 4-weeks post-MI infarction were significantly lower compared to sham-operated mice (Supporting Information Fig. S1A–S1F). Following, we performed qPCR detection of these 5 circRNAs in heart tissue at the different time points post-MI. Among the five circRNAs, circ-CELF1 exhibited the most marked upregulation in 1-day, 1-week, and 4-weeks post-MI mouse hearts compared to sham hearts (Fig. 1B). In line with this, circ-CELF1 was also significantly upregulated in CFs treated with TGF-*β*1(Fig. 1C). As such, the circ-CELF1 was selected for further investigation. Circ-CELF1 is derived from the back-splicing of the *Celf1* gene Chr2:90998580-91004862, with a length of 562 nt (Fig. 1D). RNase R treatment showed that circ-CELF1 is much more resistant to RNase R digestion compared to the linear mRNA of *Celf1* (Fig. 1E). We then assessed circ-CELF1 expression across various tissues and found the highest abundance in the heart (Fig. 1F). Furthermore, circ-CELF1 level was detected in cardiomyocytes and CFs isolated from both normal adult mice and MI model mice. Our findings revealed a significant up-regulation of circ-CELF1 level, specifically in CFs under conditions of MI (Fig. 1G). Fascinatingly, circ-CELF1 was highly enriched in 4-weeks post-MI mouse plasma compared to sham-operated mice (Fig. 1H). We subsequently detected the expression levels of circ-CELF1 in the myocardial fibrosis model induced by TAC. In concordance with our prior investigations in the MI model, our study revealed a substantial increase in circ-CELF1 levels within TAC hearts (Fig. 1I). Subcellular fractionation assay followed by qPCR revealed that circ-CELF1 was predominantly located in the cytoplasm of CFs (Fig. 1J). Along similar lines, RNA-FISH experiments demonstrated a predominant cytoplasmic localization of circ-CELF1 within the CFs (Fig. 1K).

### 3.2 Circular RNA CELF1 regulates cardiac fibrosis *in vivo*

**Figure 2:**
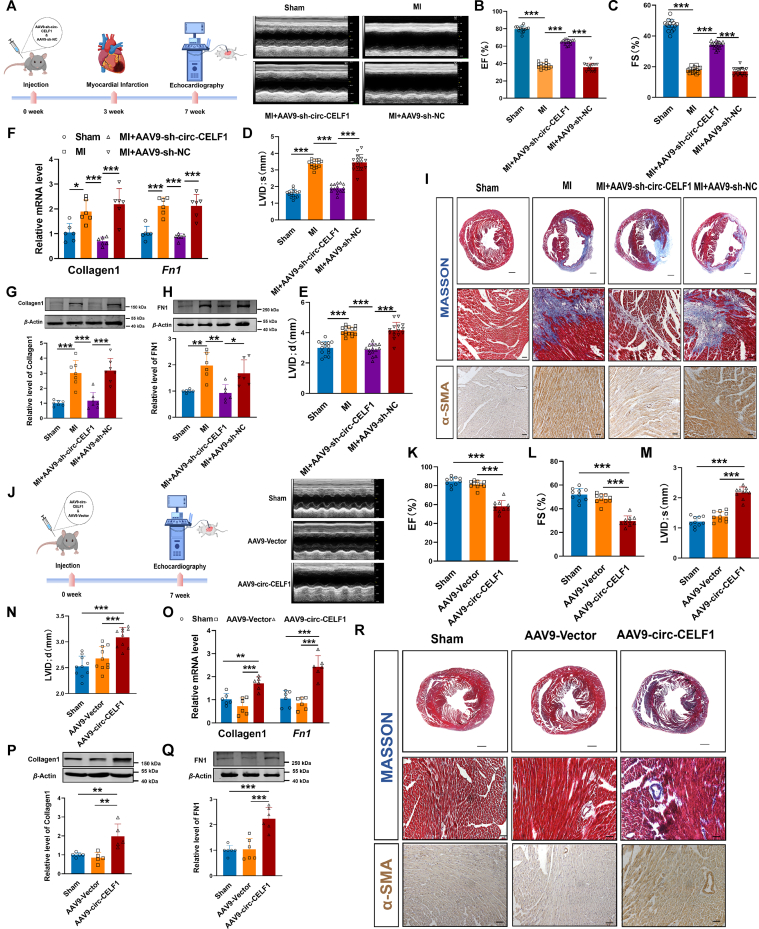
Circ-CELF1 promotes the development of cardiac fibrosis. (A) Representative echocardiography images of post-MI after circ-CELF1 knockdown. The echocardiography analysis was performed on Day 28 after MI surgery. (B–E) Echocardiographic analysis of the heart functions, including ejection fraction (EF), fractional shortening (FS), left ventricular end-systolic diameter (LVID;s), and left ventricular end-diastolic diameter (LVID;d) (*n* = 15). (F) qPCR analysis of fibrosis-related biomarkers Collagen1 and *Fn1* levels in mouse heart tissues (*n* = 6). (G, H) The levels of Collagen1 and FN1 analyzed using WB (*n* = 6–7). (I) Representative images of cardiac sections stained with Masson Trichrome (upper) and *α*-SMA expression detected *via* immunohistochemistry (lower). (J) Representative echocardiography images of circ-CELF1 upregulation. (K–N) Echocardiographic analyses of the heart functions, including EF, FS, LVID;s, LVID;d and (*n* = 10). (O) qPCR assays detecting fibrosis-related biomarkers Collagen1 and *Fn1* levels in circ-CELF1 overexpression mouse heart tissues (*n* = 6). (P, Q) Detection of Collagen1 and FN1 levels using WB (*n* = 5–6). (R) Representative images of collagen deposition detected by Masson staining (upper) and *α*-SMA expression detected by immunohistochemistry (lower). Each data point in the figure represents a unique biological replicate. The data are presented as the mean ± SD. Statistical analysis was performed with one-way ANOVA followed by Bonferroni correction. ∗*P* < 0.05, ∗∗*P* < 0.01, ∗∗∗*P* < 0.001.

To evaluate the physiological function of circ-CELF1 during MI injury, we manipulated circ-CELF1 expression using the AAV9 system driven by the periostin core promoter. The upregulation (AAV9-circ-CELF1) or silencing (AAV9-sh-circ-CELF1) of circ-CELF1 was facilitated in mouse CFs to achieve upregulation or downregulation, respectively. In brief, mice were injected *via* tail vein with AAV9-sh-circ-CELF1 or AAV9-sh-NC for 3 weeks, followed by MI for 4 weeks. Simultaneously, qPCR analysis confirmed a significant decrease in circ-CELF1 levels (Supporting Information Fig. S2A). Echocardiography analysis showed EF and FS in AAV9-sh-circ-CELF1 MI mice more than 1.5-fold higher than in AAV9-sh-NC treated MI mice (Fig. 2A–C). In the meanwhile, LVIDs and LVIDd were 30%–43% thinner in AAV9-sh-circ-CELF1 mice than in MI mice treated with AAV9-sh-NC (Fig. 2D and E). Moreover, mRNA and protein levels of fibrosis-related biomarkers were downregulated by more than 50% in the heart tissues from AAV9-sh-circ-CELF1 MI mice compared with those in AAV9-sh-NC controls (Fig. 2F–H). In addition, histological examination by Masson staining revealed that knockdown of circ-CELF1 ameliorated collagen deposition in the MI hearts compared with the AAV9-sh-NC mice. Immunohistochemical analyses revealed that knockdown of circ-CELF1 significantly reduced myofibroblast transformation in heart tissues following MI surgery, as indicated by *α*-SMA expression (Fig. 2I).

We also explored the effect of circ-CELF1 upregulation on cardiac fibrosis by administering AAV containing the full-length circ-CELF1 sequence or a control vector *via* tail vein injection for seven weeks. qPCR analysis revealed a significant increase of circ-CELF1 in mice (Fig. S2B). Echocardiography showed a 28%–39% decline in EF and FS in mice with circ-CELF1 upregulation compared to the empty vector controls (Fig. 2J–L). Meanwhile, LVIDs and LVIDd were increased in mice with circ-CELF1 upregulation compared to those treated with the empty vector (Fig. 2M and N). Consistent with this, the protein and mRNA levels of fibrosis-related biomarkers were all elevated by more than 2-fold in mice with circ-CELF1 upregulation compared to those with the empty vector construct (Fig. 2O–Q). As reflected by Masson staining and immunohistochemical analyses, the heart tissues revealed normal in vector animals, whereas circ-CELF1 upregulation resulted in collagen deposition and increased expression of *α*-SMA (Fig. 2R). Collectively, these results indicate that circ-CELF1 knockdown improved the recovery of cardiac performance after MI, while circ-CELF1 upregulation accelerated the progression of cardiac fibrosis.

### 3.3 Circular RNA CELF1 participates in cardiac fibrosis *in vitro*

**Figure 3:**
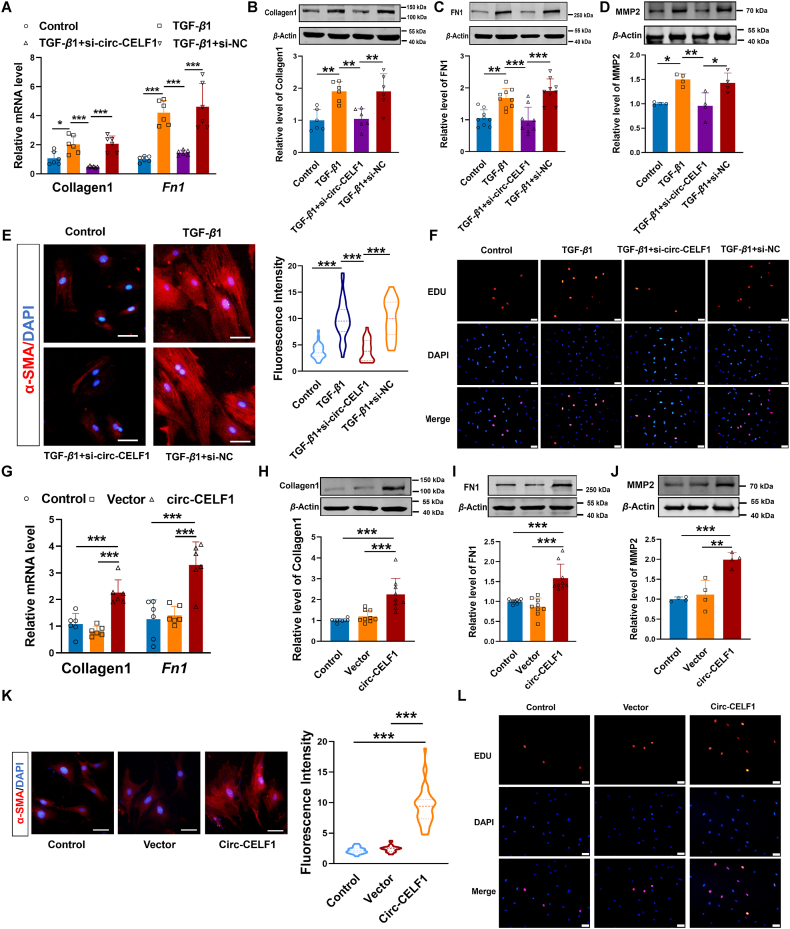
Profibrotic effects of circ-CELF1 in CFs. (A) qPCR analysis of Collagen1 and *Fn1* expression in CFs transfected with circ-CELF1 siRNA or si-NC and exposed to TGF-*β*1 for 24 h (*n* = 6). (B–D) WB analysis of Collagen1, FN1 and MMP2 expression in CFs (*n* = 4–9). (E) Immunofluorescence staining of *α*-SMA (red) in CFs, with DAPI (blue) for nuclear staining (*n* = 25–36). (F) Representative EdU staining images in CFs, with DAPI (blue) for nuclei. (G) qPCR analysis of Collagen1 and *Fn1* expression in CFs transfected with circ-CELF1 plasmid for overexpression (*n* = 6). (H–J) WB analysis of Collagen1, FN1 and MMP2 levels in CFs (*n* = 4–9). (K) Immunofluorescence staining of *α*-SMA (red) in CFs, with DAPI (blue) for nuclear staining (*n* = 27–44). (L) Representative EdU staining images in CFs, with DAPI (blue) for nuclei. Each data point in the figure represents a unique biological replicate. The data are presented as the mean ± SD. Statistical analysis was performed with one-way ANOVA followed by Bonferroni correction. ∗*P* < 0.05, ∗∗*P* < 0.01, ∗∗∗*P* < 0.001.

To further investigate the potential functions of circ-CELF1 in regulating the biological behavior of CFs, siRNAs targeting the back-splice site of circ-CELF1 were designed. The transfection efficiency revealed a notable reduction in circ-CELF1 levels within CFs (Supporting Information Fig. S3A). Silence of circ-CELF1 significantly attenuated TGF-*β*1-induced upregulation of fibrosis-related proteins and mRNA levels by 40% to 60% (Fig. 3A–C). In addition, we have performed additional experiments to investigate the process of extracellular matrix remodeling in cardiac fibrosis. Our data showed that the knockdown of circ-CELF1 resulted in a 36% reduction in MMP2 levels after TGF-*β*1 treatment (Fig. 3D). Immunostaining for *α*-SMA displayed that knockdown of circ-CELF1 resulted in a 58.6% reduction in the increase of fluorescence activity induced by TGF-*β*1 (Fig. 3E). Moreover, circ-CELF1 silencing reduced the proliferation of CFs in the presence of TGF-*β*1, as evidenced by EdU staining (Fig. 3F). Subsequently, we performed a wound healing assay to investigate the migration ability of CFs. The results demonstrated the silencing of circ-CELF1 markedly reduced the TGF-*β*1-induced enhancement of migration capacity (Fig. S3B).

Consistent with the *in vivo* functional experiments, we transfected the circ-CELF1 plasmid for overexpression, and the transfection efficiency was confirmed by a significant increase in CFs (Fig. S3C). Conversely, a greater than 1.5-fold upregulation of circ-CELF1 exacerbated fibrosis-related phenotypes at both the protein and mRNA levels (Fig. 3G–I). Simultaneously, overexpression of circ-CELF1 by more than 1.5-fold exacerbated the expression of MMP2 at protein levels (Fig. 3J). Immunostaining and EdU staining assays demonstrated that the stable circ-CELF1 upregulation boosted the proliferation ability and facilitated the differentiation of CFs into myofibroblasts by approximately 4-fold compared to the vector cells (Fig. 3K and L). Concurrently, the migration ability of CFs was markedly enhanced after overexpression of circ-CELF1 (Fig. S3D). Collectively, these findings demonstrated that circ-CELF1 promotes cardiac fibrosis *in vitro*.

Intercellular communication is one of the pivotal mechanisms regulating cardiac function and is crucial for maintaining physiological homeostasis and adapting to pathological alterations. In light of this, we assessed the effect of circ-CELF1 on the intercellular communication between cardiomyocytes and CFs using LDH and CCK8 assays. Circ-CELF1 was overexpressed in CFs, and cardiomyocytes were subsequently cultured in conditioned medium obtained from CFs overexpressing circ-CELF1(ad-circ-CELF1sup) (Fig. S3E). The LDH test results indicated that ad-circ-CELF1 medium promoted myocardial injury (Fig. S3F). Furthermore, CCK8 assay results demonstrated that ad-circ-CELF1 medium impaired the viability of cardiomyocytes (Fig. S3G). In addition, the medium containing circ-CELF1 silencing mitigated the myocardial injury of cardiomyocytes compared to TGF-*β*1-conditioned medium, as demonstrated by LDH test (Fig. S3H). Moreover, CCK8 assay displayed that knockdown of circ-CELF1 in CFs medium attenuated the impaired cardiomyocytes viability induced by TGF-*β*1-conditioned medium from CFs (Fig. S3I). These results suggest that circ-CELF1 may influence cardiomyocytes through the promotion of secretion of relevant molecules from CFs.

Additionally, we investigated whether human circ-CELF1 (hsa-circ-CELF1) exhibits the same profibrotic property in HCFs as the mouse circ-CELF1. Sanger sequencing confirmed the junction site of hsa-circ-CELF1 (Supporting Information Fig. S4A). The resistance of hsa-circ-CELF1 to RNase R digestion exhibited a significantly higher level compared to the linear mRNA of *hsa-CELF1* (Fig. S4B). Consistent with mouse circ-CELF1, hsa-circ-CELF1 was also upregulated in HCFs treated with TGF-*β*1(Fig. S4C). The qPCR analysis revealed that the knockdown of hsa-circ-CELF1 resulted in the downregulation of *hsa-CELF1* mRNA (Fig. S4D and S4E). Silence of hsa-circ-CELF1 attenuated TGF-*β*1-induced upregulation of fibrosis-related proteins and mRNA levels in HCFs (Fig. S4F–S4H). EdU staining assays demonstrated that hsa-circ-CELF1 silencing reduced the proliferation of CFs in the presence of TGF-*β*1 (Fig. S4I). This indicated that circ-CELF1 potentially acts as a potential novel therapeutic target for cardiac fibrosis.

To further demonstrate the infectivity of AAV9 using a periostin core promoter into fibroblast was efficient, we isolated cardiomyocytes and fibroblasts from adult mice and evaluated the relative infectivity by measuring circ-CELF1 levels in both cell fractions. Our findings revealed a significant down-regulation of circ-CELF1 levels, specifically in CFs, but not in cardiomyocytes. These results indicate that the knockdown of circ-CELF1 is specific to cardiac fibroblasts (Fig. S4J and S4K). Additionally, mice were intravenously injected *via* tail vein with AAV9-sh-circ-CELF1 or AAV9-sh-NC for 3 weeks, followed by MI for 4 weeks and isolated cardiac fibroblasts to evaluate the physiological role of circ-CELF1. The protein and mRNA levels of fibrosis-related biomarkers were all downregulated in cardiac fibroblasts isolated from AAV9-sh-circ-CELF1 treated MI mice compared to those from MI mice (Fig. S4L–S4N). Furthermore, immunostaining for *α*-SMA revealed a significant reduction in fluorescence intensity in cardiac fibroblasts isolated from AAV9-sh-circ-CELF1 MI mice, as compared to those isolated from MI mice (Fig. S4O).

### 3.4 Identification of BRPF3 as the target of circular RNA CELF1 in CFs

**Figure 4:**
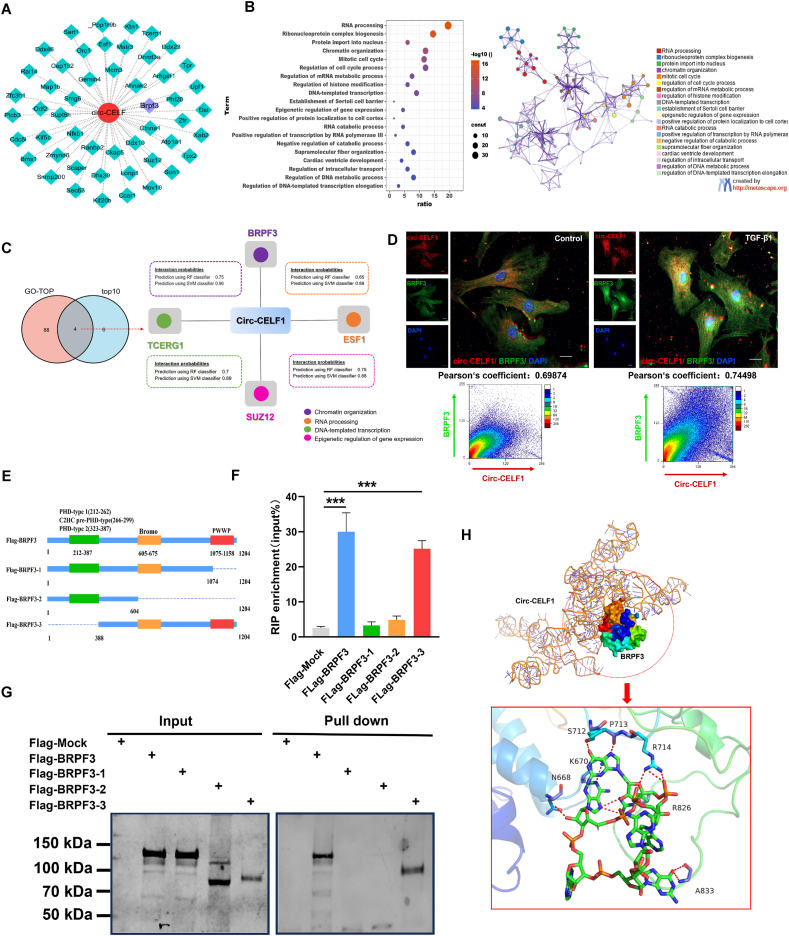
Circ-CELF1 directly binds to BRPF3. (A) Screening of circ-CELF1 downstream targets using the CatRAPID database. (B) GO enrichment analysis of pathways correlated with circ-CELF1-mediated proteins. (C) Venn diagram illustrating circ-CELF1's downstream target BRPF3. (D) Colocalization of circ-CELF1 and BRPF3 was investigated in both normal CFs and TGF-*β*1-treated CFs, with circ-CELF1 labeled in Cy3 (red), BRPF3 in 488R (green), and nuclei stained with DAPI (blue). (E) Design of flag-tagged BRPF3 truncation expression plasmids. (F) RIP assay in HEK293T cells transfected with BRPF3 truncation vectors to validate the binding domain (*n* = 4). (G) RNA pull-down assay in CFs transfected with wild-type and truncated BRPF3 expression plasmids, using biotin-labeled oligos or circ-CELF1 probes. (H) Molecular docking of circ-CELF1 interaction with the PWWP domain (1075–1158aa) of BRPF3. Each data point in the figure represents a unique biological replicate. The data are presented as the mean ± SD. Statistical analysis was performed with one-way ANOVA followed by Bonferroni correction. ∗∗∗*P* < 0.001.

To investigate the precise mechanism underlying circ-CELF1 in cardiac fibrosis, bioinformatics analysis by the database of CatRAPID was performed to elucidate the potential proteins binding to the circ-CELF1 (Fig. 4A). We conducted an intersection analysis of the CatRAPID prediction results (TOP10) and GO enrichment analysis (TOP10) of the most correlated pathway among the circ-CELF1-regulated proteins. Strikingly, we found four protein molecules: BRPF3, TCERG1, ESF1and SUZ12 (Fig. 4B and C). Among them, the BRPF3 had the highest score based on sequence coverage. To further confirm these findings, the IF co-staining assay revealed that the colocalization of circ-CELF1 and BRPF3 both in control group and TGF-*β*1 induced cardiac fibrosis model group, providing evidence for the interaction (Fig. 4D). To precisely determine the interaction site between circ-CELF1 and BRPF3, the online database UniProt was used to construct Flag-tagged full-length BRPF3 and a series of Flag-tagged BRPF3 truncation mutants according to the BRPF3 function domains for RIP and RNA-pulldown assays (Fig. 4E). RIP assay showed that the PWWP domain (1075–1158aa) of BRPF3, but not other domains, was crucial for the interaction with circ-CELF1 (Fig. 4F). Furthermore, the RNA-pulldown assay demonstrated that the biotinylated circ-CELF1 probe specifically captured the 1075–1158 amino acid region of BRPF3 (Fig. 4G). The molecular docking analysis revealed that circ-CELF1 formed bond interactions with the PWWP domain (1075–1158aa) of BRPF3 (Fig. 4H). Altogether, these conclusions confirm the PWWP domain (1075–1158aa) of BRPF3 is essential for its interaction with circ-CELF1.

### 3.5 BRPF3 regulates cardiac fibrosis *in vitro*

**Figure 5:**
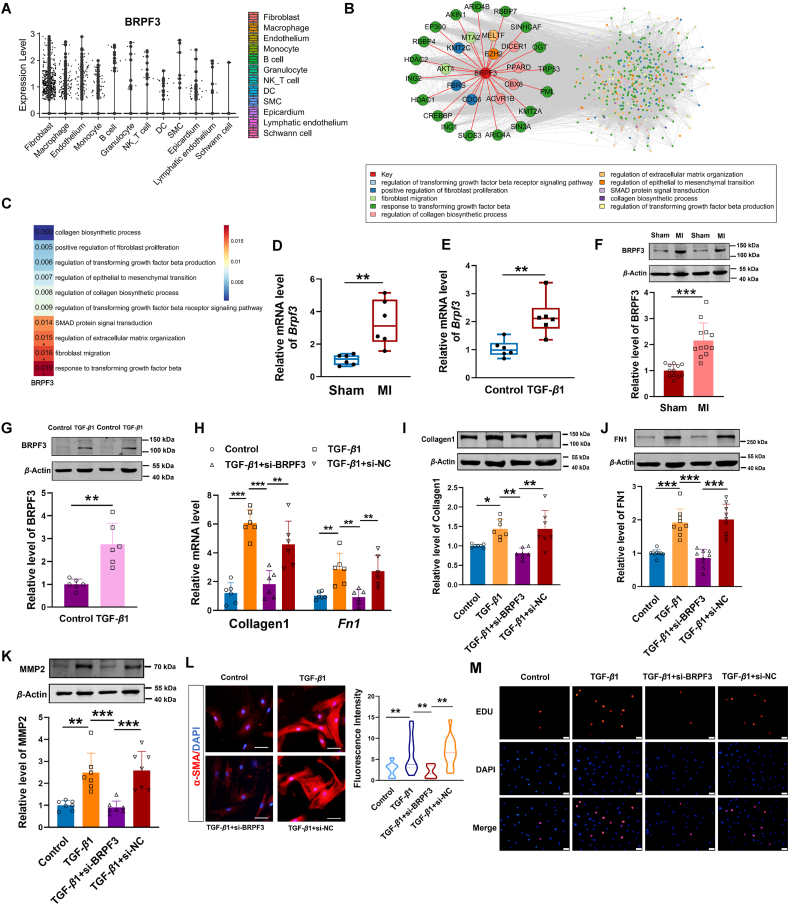
Silencing BRPF3 reverses cardiac fibrosis *in vitro*. (A) The analysis on the expression level of BRPF3 in various cardiac cell types, including fibroblasts, macrophages, and endothelial cells in a single-cell RNA-seq dataset. (B) Protein–protein interaction network elucidates the role of BRPF3 in the fibrosis regulatory network. (C) Correlation analysis of BRPF3 expression with fibrosis-related pathway activity scores in cardiac fibroblasts from myocardial infarction scRNA-seq data (E-MTAB-7895). The color bar represents the strength of correlation, ranging from −1 (perfect negative correlation, blue) to +1 (perfect positive correlation, red), with statistical significance indicated by asterisks (∗*P* < 0.05, ∗∗*P* < 0.01, ∗∗∗*P* < 0.001). Pathway activity scores were calculated using the AddModuleScore function from Seurat based on fibrosis-related pathways. (D, E) qPCR analysis of *Brpf3* mRNA expression in MI hearts and TGF-*β*1-treated CFs (*n* = 6). (F, G) WB analysis of BRPF3 protein levels in MI hearts and TGF-*β*1-treated CFs (*n* = 6–12). (H) qPCR analysis of Collagen1 and *Fn1* levels in CFs transfected with BRPF3 siRNA or si-NC, followed by TGF-*β*1 exposure for 24 h (*n* = 6). (I–K) WB analysis of Collagen1, FN1 and MMP2 levels in CFs (*n* = 7–9). (L) Immunofluorescence staining of *α*-SMA in CFs. Scale bars = 100 μm, *α*-SMA (red), nuclei (blue) (*n* = 9–20). (M) Representative images of EdU staining in CFs, with nuclei stained by DAPI (blue). Scale bars = 100 μm. Each data point in the figure represents a unique biological replicate. The data are presented as the mean ± SD. Statistical analysis was performed with one-way ANOVA followed by Bonferroni correction and Student's *t* test for two means. ∗*P* < 0.05, ∗∗*P* < 0.01, ∗∗∗*P* < 0.001.

To discuss the position of BRPF3 in the cardiac fibrosis, we analyzed data from a single-cell RNA-seq dataset (E-MTAB-7895) obtained from a public database^27^. A comprehensive analysis of the BRPF3 expression levels in various cardiac cell types, including fibroblasts, macrophages, and endothelial cells, surprisingly revealed a significant enrichment of BRPF3 in the fibroblasts (Fig. 5A). Subsequently, we identified pathway-associated genes and constructed a fibrosis regulatory network to analyze the position of BRPF3 within this network (Fig. 5B). By calculating the single-cell fibroblast data from mice 28 days post-myocardial infarction, a positive correlation was identified between BRPF3 expression and fibrosis-related pathways. Among those pathways, the “regulation of extracellular matrix organization”, “fibroblast migration” and “response to transforming growth factor beta” exhibited a significantly positively correlated with BRPF3 expression. These findings indicate that BRPF3 plays a central position in the fibrosis regulatory network and exhibits synergistic interactions with multiple fibrosis signaling pathways (Fig. 5C). To further evaluate the function of BRPF3, qPCR and WB assay were applied to examine the expression of BRPF3 in CFs and mouse heart tissue. The qPCR analysis revealed a significant up-regulation of *Brpf3* mRNA levels in CFs exposed to TGF-*β*1 and in the myocardial tissue of mice with MI (Fig. 5D and E). In line with the qPCR experiments described above, the protein levels of BRPF3 were also upregulated in both TGF-*β*1-treated CFs and in MI mice (Fig. 5F and G), indicating its potential role in promoting cardiac fibrosis. To investigate the function of BRPF3 in regulating the biological behavior of cardiac fibrosis, siRNAs for *Brpf3* were designed to knock down the endogenous *Brpf3*. qPCR assays confirmed that the transfection of the siRNA-3 significantly reduced *Brpf3* expression (Supporting Information Fig. S5A). The inhibition of BRPF3 attenuated TGF-*β*1-induced upregulation of fibrosis-related mRNA and protein levels (Fig. 5H–J). We further observed that knockdown of BRPF3 mitigated the upregulation of MMP2 levels induced by TGF-*β*1 in CFs (Fig. 5K). In addition, immunostaining for α-SMA revealed that knockdown of BRPF3 mitigated the upregulation of fluorescence activity induced by TGF-*β*1 (Fig. 5L). Moreover, the silencing of BRPF3 attenuated the proliferation of CFs in the presence of TGF-*β*1, as demonstrated by EdU staining (Fig. 5M). Moreover, wound healing assays demonstrated a significant reduction in migration ability after BRPF3 knockdown in CFs in the presence of TGF-*β*1(Fig. S5B). Together, the above data demonstrate that BRPF3 promotes cardiac fibrosis.

### 3.6 Circular RNA CELF1 accelerates cardiac fibrosis progression by inhibiting BRPF3 degradation *via* the ubiquitin–proteasomal pathway

**Figure 6:**
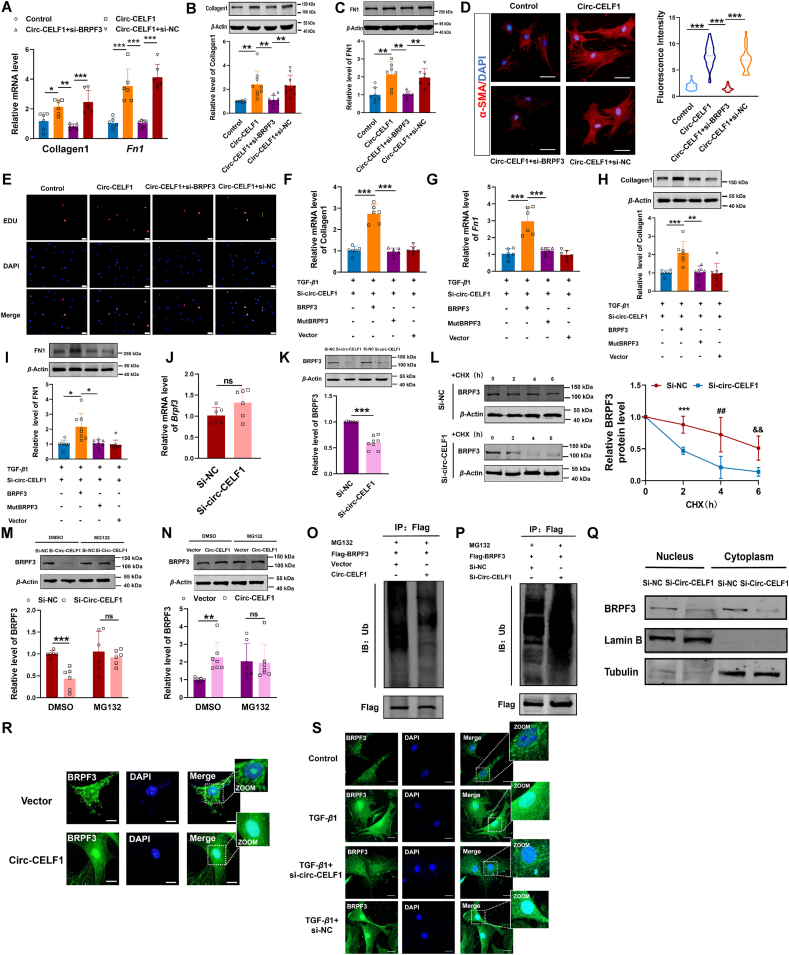
Circ-CELF1 enhances BRPF3 expression in the nucleus by suppressing the ubiquitin–proteasomal degradation pathway. (A) qPCR showing Collagen1 and *Fn1* levels in CFs. CFs were co-transfected with circ-CELF1 overexpression plasmid and BRPF3 siRNA (*n* = 6). (B, C) The WB assays revealed the expression levels of Collagen1 and FN1 in CFs (*n* = 7–9). (D) The immunofluorescence staining of *α*-SMA in CFs. Scale bars represent 100 μm. Cells were stained for *α*-SMA (red) and nuclei with DAPI (blue) (*n* = 22–39). (E) Representative images of EdU staining in CFs. The nuclei are stained by DAPI (blue). (F, G) The qPCR analysis revealed the levels of Collagen1 and *Fn1* in CFs. CFs were transfected with a BRPF3 overexpression plasmid, circ-CELF1 siRNA, and a truncated mutant of BRPF3, respectively, followed by exposure to TGF-*β*1 for 24 h (*n* = 6). (H, I) The levels of Collagen1 and FN1 in CFs were assessed using WB assays (*n* = 7–8). (J) qPCR assays were performed to evaluate the mRNA expression of *Brpf3* in CFs with circ-CELF1 knockdown (*n* = 6). (K) WB showed the protein level of BRPF3 in circ-CELF1-silenced CFs (*n* = 8). (L) WB analysis of BRPF3 protein stability in CFs treated with cycloheximide (CHX). CFs were transfected with circ-CELF1 siRNA or si-NC and treated with CHX (50 μg/mL) for 0, 2, 4, or 6 h (*n* = 5). (M, N) Circ-CELF1-silenced (M) or upregulated (N) CFs were treated with or without MG-132 (50 μmol/L), and BRPF3 expression was analyzed by WB (*n* = 6–7). (O, P) Ubiquitination assays revealed BRPF3 ubiquitination levels in CFs with circ-CELF1 upregulation (O) or silencing (P). The CFs were pretreated with MG132 (50 μmol/L). (Q) The nuclear expression of BRPF3 was examined by WB after silencing circ-CELF1 in CFs. (R) The nuclear expression of BRPF3 was examined by immunofluorescence staining after overexpressing circ-CELF1 in CFs. Scale bars represent 100 μm. Cells stained for BRPF3 (green) and nuclei with DAPI (blue). (S) The nuclear localization of BRPF3 was assessed through immunofluorescence staining following a 24 h exposure of CFs to TGF-*β*1. Scale bars represent 100 μm. Cells stained for BRPF3 (green) with DAPI (blue) for nuclei. Each data point in the figure represents a unique biological replicate. The data are presented as the mean ± SD. Statistical analysis was performed with one-way ANOVA followed by Bonferroni correction and Student's *t* test for two means. ∗*P* < 0.05, ∗∗*P* < 0.01, ∗∗∗*P* < 0.001, ^##^*P* < 0.01, ^&&^*P* < 0.01.

We then examined the functional relationship between circ-CELF1 and BRPF3 in CFs. The qPCR and WB assay demonstrated that circ-CELF1 upregulation increased fibrotic markers responses, whereas these effects were reversed upon knockdown of BRPF3 (Fig. 6A–C). Similarly, immunostaining for *α*-SMA analyses and EdU staining indicated that the differentiation and proliferation of CFs induced by circ-CELF1 could be rescued by silencing BRPF3 (Fig. 6D and E). Meanwhile, qPCR and WB assay revealed that the wild-type BRPF3 plasmid reversed fibrotic markers responses caused by circ-CELF1 knockdown, whereas the mutant-type BRPF3 plasmid (with a mutation in the PWWP domain at amino acids 1075-1158) did not demonstrate such reversal effects in the presence of TGF-*β*1 (Fig. 6F–I). Moreover, we examined the specific regulatory mechanism of circ-CELF1 on BRPF3 expression in CFs, qPCR and WB results showed that circ-CELF1 knockdown decreased the protein levels of BRPF3 without notable effects on the mRNA levels of *Brpf3* (Fig. 6J and K). Conversely, circ-CELF1 overexpression led to a significant upregulation in the protein levels of BRPF3 (Fig. S5C). Simultaneously, the protein levels of BRPF3 were downregulated in the heart tissues from AAV9-sh-circ-CELF1 MI mice compared to those in AAV9-sh-NC control mice (Fig. S5D). The findings suggest that circ-CELF1 regulates BRPF3 expression post-transcriptionally. CFs were treated with cycloheximide (CHX) to assess the impact of circ-CELF1 on BRPF3 protein stability. The findings revealed a significant increase in the degradation rate of the BRPF3 protein upon silencing circ-CELF1 in CFs (Fig. 6L). MG132, a proteasome inhibitor, effectively rescued BRPF3 degradation induced by circ-CELF1 knockdown in CFs (Fig. 6M). Meanwhile, circ-CELF1 upregulation in CFs resulted in an accumulation of BRPF3; however, treatment with MG132 counteracted the circ-CELF1-induced alterations in BRPF3 (Fig. 6N). The findings suggest that circ-CELF1 modulates the degradation pathway of the BRPF3 protein. The ubiquitination process represents a prevalent degradation pathway for protein molecules^28^. Correspondingly, CFs were treated with MG132, and circ-CELF1 upregulation inhibited the ubiquitination of BRPF3 (Fig. 6O). Additionally, silencing circ-CELF1 enhanced the ubiquitination of BRPF3 in response to MG132 treatment (Fig. 6P). The above evidence substantiated that the interaction between circ-CELF1 and BRPF3 enhances its stability, consequently resulting in sustained elevation of protein expression. Subsequently, we investigated whether circ-CELF1 affects the subcellular location of the BRPF3 protein. We observed that silencing circ-CELF1 repressed BRPF3 expression in the nucleus (Fig. 6Q), whereas circ-CELF1 upregulation promoted the nucleus translocation of the BRPF3 protein (Fig. 6R). Furthermore, we observed that TGF-*β*1 promoted the nuclear translocation of the BRPF3 protein in CFs, while silencing circ-CELF1 repressed BRPF3 expression in the nucleus (Fig. 6S). Taken together, our findings demonstrate that circ-CELF1 exerts an inhibitory effect on the ubiquitination-degradation of BRPF3, thereby facilitating its nuclear translocation and influencing the progression of cardiac fibrosis.

### 3.7 BRPF3–KAT7 axis regulates Celf1 transcription and cardiac fibrosis *via* H3K14ac

**Figure 7:**
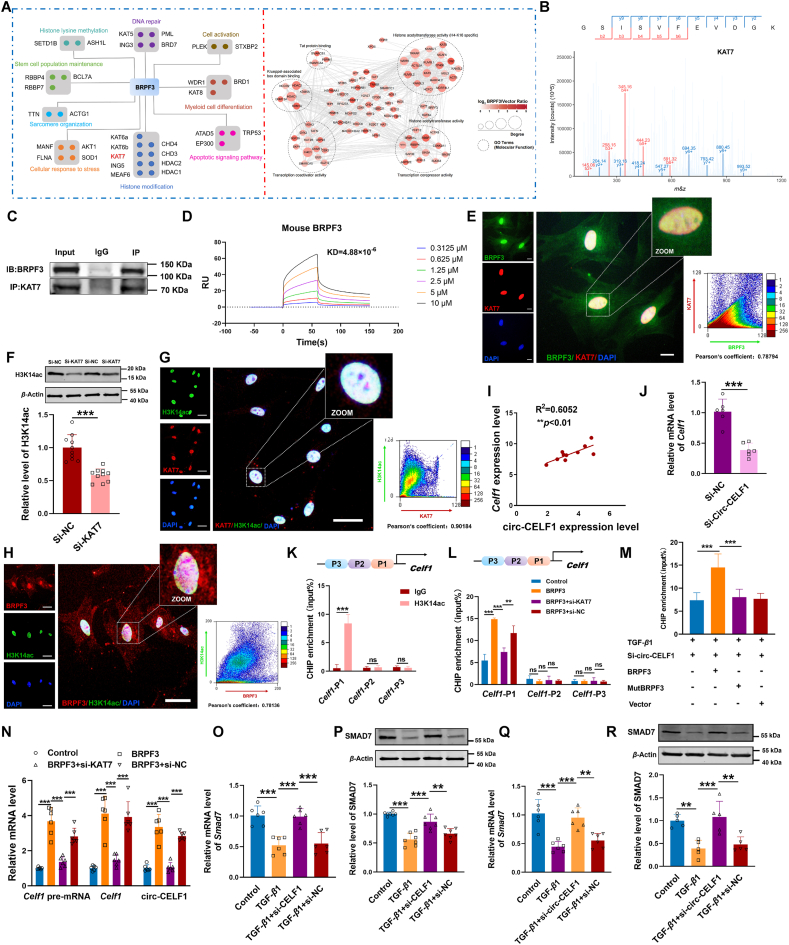
BRPF3-mediated feedback loop in TGF-*β*1-treated CFs. (A) Identification of downstream targets of BRPF3 using the STRING database and co-immunoprecipitation (Co-IP) experiments coupled with 4D-FastDIA LC–MS/MS analysis. (B) A mass spectrometer was used to detect the KAT7 interacting with BRPF3. (C) Co-IP and WB analysis were used to evaluate the interactions of BRPF3 and KAT7 in mouse heart tissue. (D) Recombinant KAT7 proteins were immobilized on the CHIPs. The binding between KAT7 and BRPF3 (0.3125, 0.625, 1.25, 2.5, 5 and 10 μmol/L, from the bottom to top) was assessed by SPR. (E) Colocalization of BRPF3 (green) and KAT7 (red) in TGF-*β*1-treated CFs, with DAPI (blue) for nuclear staining. (F) WB analysis of H3K14ac expression in KAT7-silenced CFs (*n* = 10). (G, H) Colocalization of H3K14ac (green) with KAT7 (red) and BRPF3 (red) in TGF-*β*1-treated CFs, with DAPI (blue). (I) Correlation between circ-CELF1 and *Celf1* mRNA levels in MI mice *via* qPCR (*n* = 10). (J) qPCR analysis of *Celf1* mRNA levels in circ-CELF1-silenced CFs (*n* = 6). (K, L) CHIP qPCR assays measuring H3K14ac levels in *Celf1* promoter regions in CFs with BRPF3 overexpression or KAT7 silencing (*n* = 4–6). (M) CHIP qPCR for H3K14ac in *Celf1* promoters after co-transfection with BRPF3 plasmid, circ-CELF1 siRNA, and BRPF3 mutant (*n* = 6). (N) qPCR showing *Celf1* pre-mRNA, *Celf1* mRNA, and circ-CELF1 expression in CFs co-transfected with BRPF3 plasmid and KAT7 siRNA (*n* = 6). (O–R) qPCR and WB of SMAD7 mRNA and protein levels in CFs transfected with *Celf1* or circ-CELF1siRNA and exposed to TGF-*β*1 (*n* = 5–7). Each data point in the figure represents a unique biological replicate. The data are presented as the mean ± SD. Statistical analysis was performed with one-way ANOVA followed by Bonferroni correction and Student's *t* test for two means. ∗∗*P* < 0.01, ∗∗∗*P* < 0.001.

To further investigate the underlying mechanism by which BRPF3 regulates cardiac fibrosis, we used the STRING database to predict and score the proteins that potentially interact with BRPF3. Meanwhile, to identify proteins that interact with BRPF3, we also conducted co-immunoprecipitation (Co-IP) experiments coupled with 4D-FastDIA LC–MS/MS analysis (Fig. 7A). BRPF3 functions as a scaffold in the histone acetyltransferase complex, playing crucial roles in gene regulation and development. Consistent with previous research, GO analysis revealed that the binding proteins of BRPF3 are predominantly enriched in histone acetyltransferase activity (Supporting Information Fig. S6A). Among the predicted proteins, KAT7, EAF6, and ING5 achieved the highest score, indicating the highest credibility in interacting with BRPF3. The Co-IP assay further confirmed that the specific interaction between KAT7 and BRPF3 was highly significant (Fig. S6B). To further substantiate the interaction between BRPF3 and KAT7 in CFs, we performed IP experiments followed by mass spectrometric analysis. As illustrated in Fig. 7B, the results confirm that BRPF3 interacted with the KAT7 protein in CFs. The Co-IP assay in mouse heart tissue to further validate the specific interaction between KAT7 and BRPF3 (Fig. 7C). Surface plasmon resonance (SPR) analysis revealed BRPF3 binds to the recombinant KAT7 protein with high affinity, exhibiting a dissociation constant (*K*_D_) of 4.88 × 10^−6^ (Fig. 7D). Additionally, we used protein–protein docking analysis to further verify the interaction between BRPF3 and KAT7 and observed a higher binding affinity between BRPF3 and KAT7 (Fig. S6C). Moreover, immunofluorescence results demonstrated the colocalization of BRPF3 and KAT7 in the nuclei of CFs upon TGF-*β*1 stimulation (Fig. 7E). Further investigation revealed that the overexpression of circ-CELF1 resulted in a significant upregulation in the protein levels of KAT7 (Fig. S6D). GO analysis showed that the functional enrichment of KAT7 was predominantly involved in the process of histone acetylation modification (Fig. S6E). The KAT7 protein is known to form two distinct histone acetyltransferase (HAT) complexes, each specifically acetylating either H3 or H4^29^. Therefore, we investigated the acetylation status of H3 and H4 tails in CFs with KAT7 knockdown. Among KAT7's potential acetylation sites, only the acetylation of H3K14 was inhibited, while other sites remained unaffected (Fig. 7F, Fig. S6F–S6I). In addition, the fluorescence intensity of H3K14ac was significantly reduced in CFs upon knockdown of KAT7, as compared to the negative control (Fig. S6J). Subsequently, immunofluorescence confirmed the colocalization of KAT7 and H3K14ac in the nucleus of CFs induced by TGF-*β*1 (Fig. 7G). Simultaneously, it was observed that the colocalization between BRPF3 and H3K14ac in the nucleus of CFs responsive to TGF-*β*1 stimulation (Fig. 7H).

In light of this, we subsequently elucidated the mechanism by which KAT7 regulates the transcription of specific genes implicated in cardiac fibrosis. Surprisingly, a positive correlation was observed between circ-CELF1 and *Celf1* mRNA levels (Fig. 7I). qPCR analysis revealed that the knockdown of circ-CELF1 resulted in the downregulation of *Celf1* mRNA expression (Fig. 7J). These findings motivated us to explore whether there is transcriptional regulation between KAT7 and *Celf1* expression, thereby establishing a feedback loop in circ-CELF1 production. Therefore, we then focused on elucidating the role of KAT7 in the regulation of *Celf1* transcription. We initially searched the *Celf1* promoter in the UCSC database and identified histone acetylation modification at the specific promoter region. Furthermore, we divided the promoter region of *Celf1* into three segments, which indicated the potential enrichment of H3K14ac acetylation. CHIP assays were performed using H3K14ac histone antibodies, revealing the detection of H3K14ac acetylation in the first *Celf1* site within the promoter region (Fig. 7K). Additionally, BRPF3 increased the level of H3K14ac in *Celf1* gene promoters, whereas the knockdown of KAT7 attenuated this effect (Fig. 7L). Meanwhile, CHIP assays revealed that the wild-type BRPF3 plasmid markedly enhanced the level of H3K14ac at the promoters of the *Celf1* gene relative to circ-CELF1 knockdown in the presence of TGF-*β*1. Conversely, the mutant-type BRPF3 plasmid (with a mutation in the PWWP domain at amino acids 1075-1158) did not exhibit a comparable effect under the same conditions (Fig. 7M). These findings support the role of BRPF3 as a modular scaffold that enhances H3K14ac acetylation in the *Celf1* promoter by recruiting KAT7.

To further explore the potential roles of CELF1 in regulating the biological behavior of CFs, we systematically analyzed the expression levels of CELF1 in CFs and mouse heart tissue. The analysis revealed a significant up-regulation of *Celf1* mRNA levels in the myocardial tissue of mice subjected to MI as well as in CFs treated with TGF-*β*1 (Supporting Information Fig. S7A and S7B). Subsequently, we transfected *Celf1* siRNA into CFs to silence endogenous CELF1 and identified the effect of CELF1 knockdown on cardiac fibrosis. The results showed that the silencing of CELF1 reduced the TGF-*β*1-induced upregulation of fibrosis-associated proteins and mRNA levels (Fig. S7C–S7F). In addition, EdU staining displayed that knockdown of CELF1 attenuated the proliferation ability induced by TGF-*β*1 (Fig. S7G). In accordance with the findings reported in the published article^30^, CELF1 also exerts a catalytic influence on cardiac fibrosis. We then examined the functional relationship between CELF1 and circ-CELF1 in CFs. The qPCR and WB assay demonstrated that knockdown of CELF1 resulted in the downregulation of TGF-*β*1-induced fibrotic marker responses, whereas these effects were reversed upon overexpression of circ-CELF1 (Fig. S7H–S7J). Similarly, EdU staining indicated that the proliferation of CFs induced by TGF-*β*1 could be attenuated by silencing CELF1, whereas overexpression of circ-CELF1 exacerbated the fibrosis-related phenotype(Fig. S7K). Taken together, these findings provide evidence that CELF1 modulates cardiac fibrosis *via* the circ-CELF1.

The production of circRNA is known to occur through alternative splicing of pre-mRNA. qPCR assays revealed knockdown of KAT7 significantly reduced BRPF3 induced *Celf1* pre-mRNA, *Celf1* mRNA and circ-CELF1 expression (Fig. 7N). These findings suggest that BRPF3 is involved in the transcription and formation of circ-CELF1, cooperating with KAT7.

To further elucidate the upstream regulatory mechanism of CELF1, the UCSC Genome Browser database was employed to investigate the primary transcriptional factor of *Celf1*. Sixteen transcription factors were identified from the UCSC database based on their transcription direction and score. Subsequently, an intersection analysis with the JASPAR database resulted in the final identification of eight transcription factors. Ultimately, SOX10 and NFATC1 were selected based on the quantity and score of their binding sites. Additionally, to elucidate whether SOX10 and NFATC1 directly regulates the promoter of *Celf1*, chromatin immunoprecipitation followed by quantitative PCR (CHIP-qPCR) was employed. The SOX10 antibody did not significantly enrich the *Celf1* promoter in CFs, whereas NFATC1 antibody exhibited pronounced enrichment of the *Celf1* promoter. The percentage of *Celf1* promoter enrichment with the SOX10 antibody was significantly lower, ranging from 0.47% to 0.89%, compared to the Input. This is notably less than the enrichment observed with the NFATC1 antibody, which accounts for 5.62%–8.88% of the Input content (Supporting Information Fig. S8A). Furthermore, the NFATC1 antibody was used in CHIP-qPCR assays, and results revealed that knockdown of KAT7 significantly reduced BRPF3-induced *Celf1* transcriptional activation (Fig. S8B). The findings suggested that NFATC1 functions as a transcription factor for *Celf1*. To further investigate the expression regulation mechanism of circ-CELF1. Alternative splicing plays a crucial role in the development and progression of cardiovascular diseases^31^. As circRNAs are generated through the alternative splicing of pre-mRNA, splicing factors interact with the RNA sequences flanking the circ-CELF1 sequence on the pre-mRNA strand, thereby facilitating the splicing process and enhancing the expression of circ-CELF1. Therefore, we used the RBPmap database to predict and score the potential splicing factors that may bind to the introns flanking exons 3 and 8 of *Celf1* pre-mRNA. Among the predicted splicing factors, KHSRP, SF1, SFPQ and U2AF2 were found to bind to the introns flanking exons 3 and 8 of CELF1 pre-mRNA and achieved notably high binding scores. This suggests a high degree of confidence in their interaction with circ-CELF1. Subsequently, we performed qPCR to detect the expression levels of these four splicing factors in a cellular model of myocardial fibrosis. Among the four splicing factors, *U2af2* was upregulated in CFs treated with TGF-*β*1 (Fig. S8C). To further confirm the interaction between circ-CELF1 and U2AF2, qPCR assays were conducted. Upon knockdown of U2AF2 in CFs, a significant decrease in the expression level of circ-CELF1 was observed (Fig. S8D and S8E). Taken together, these findings indicate that U2AF2 may modulate the expression of circ-CELF1 through alternative splicing.

Previous studies have demonstrated that silencing CELF1 leads to an increase in SMAD7 expression, thereby inhibiting the pathogenesis of liver fibrosis^30^. Therefore, we further explored the effect of CELF1 on SMAD7 expression in CFs. As depicted in Fig. 7O and P, TGF-*β*1 decreased the mRNA and protein levels of SMAD7. On the other hand, the knockdown of CELF1 elevated the mRNA and protein levels of SMAD7. Moreover, silencing circ-CELF1 reversed TGF-*β*1's effects on SMAD7 (Fig. 7Q and R). The overexpression experiment of circ-CELF1 in CFs resulted in a downregulation of SMAD7 levels. Conversely, co-transfection with BRPF3 siRNA led to an upregulation of SMAD7 expression at both the mRNA and protein levels (Supporting Information Fig. S9A and S9B). Meanwhile, qPCR and WB assay demonstrated that upregulation of BRPF3 decreased SMAD7 expression, whereas these effects were abrogated upon knockdown of KAT7 (Fig. S9C and S9D). *In vivo* experiments demonstrate that the protein levels of SMAD7 were downregulated in the heart tissues from MI mice compared to those in sham mice. Conversely, knockdown of circ-CELF1 reversed the downregulation of SMAD7 protein expression observed in MI mice (Fig. S9E). The BRPF3 complex engages the histone acetyltransferase KAT7, which acts as a scaffold to increase the amount of H3K14ac in the *Celf1* promoter regions and decrease the expression of SMAD7, driving cardiac fibrosis. Previous studies have demonstrated that silencing CUGBP1(also known as CELF1) in LX-2 cells leads to increased expression of IFN-*γ* and subsequently upregulates the expression of SMAD7, a downstream target of the IFN-*γ* signaling pathway^30^. We used siRNA to knockdown *Celf1* in CFs and subsequent qPCR analysis confirmed that siRNA-*Celf1* increased the expression level of IFN-*γ* mRNA (Fig. S9F). Subsequently, we investigated whether CELF1 influences the stability of IFN-*γ* mRNA. CFs were treated with the transcription inhibitor actinomycin D. This treatment resulted in a time-dependent decay of IFN-*γ* mRNA. Notably, knockdown of CELF1 inhibited this decay process (Fig. S9G). Therefore, inhibiting CELF1 to enhance IFN-*γ* signaling also contributes to the mechanism of cardiac fibrosis and is involved in the BRPF3/KAT7 axis.

## 4 Discussion

**Figure 8:**
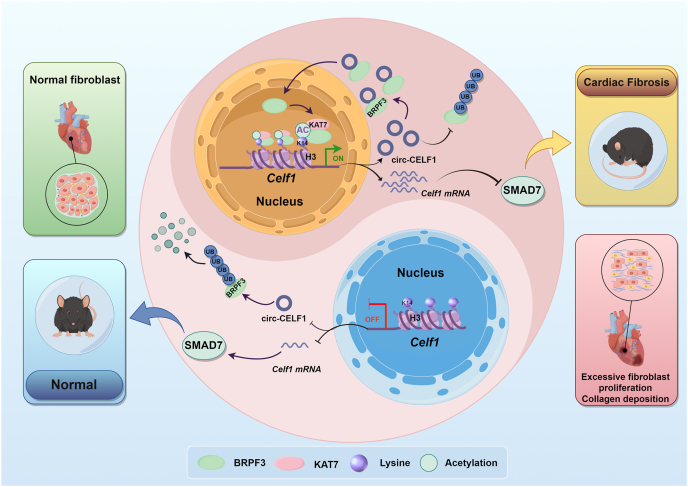
The schematic diagram illustrates a novel feedback loop involving CELF1/circ-CELF1/BRPF3/KAT7 in cardiac fibrosis pathogenesis.

Several studies have previously reported that circRNAs with various activities may modulate signaling pathways as well as targets associated with the etiology of cardiac fibrosis. This study identified circular RNA (circ-CELF1) through dataset screening and validation in pathological samples. Notably, the expression was upregulated in both *in vivo* models of MI induced by ligation of the left anterior descending coronary artery and *in vitro* models stimulated with TGF-*β*1. Consequently, our investigation was primarily focused on elucidating the role and potential mechanism underlying the involvement of circ-CELF1 in cardiac fibrosis. Mechanistically, circ-CELF1 interacted with BRPF3 to inhibit BRPF3 ubiquitination and degradation, thereby boosting the translocation of BRPF3 into the cell nucleus and recruitment of KAT7. This ultimately led to an increase in H3K14 acetylation levels within *Celf1* promoter regions. Importantly, CELF1 not only suppressed the subsequent response of the downstream target gene SMAD7 expression to promote cardiac fibrosis but also facilitated *Celf1* pre-mRNA transcription and back-splicing to facilitate circ-CELF1 biogenesis. These molecules establish a feedback loop for circ-CELF1 production and enhance cardiac fibrosis (Fig. 8).

Circ-CELF1 is a recently discovered circRNA containing the exon sequence of the *Celf1* gene. A recent study found that circ-CELF1 inhibits myocardial fibrosis by regulating the expression of DKK2 through FTO/m6A and miR-636^32^. Conversely, our studies demonstrated that circ-CELF1 exhibited pro-fibrotic properties. This contradiction deserves further exploration and analysis. As circRNAs are generated *via* the alternative splicing of pre-mRNA, the observed discrepancy may be attributed to variations in splicing sites. The same gene is capable of generating multiple circular RNAs through alternative splicing sites and a variety of splicing mechanisms. CircRNAs derived from the same parental gene may exhibit distinct biological functions, and the specific isoforms can display antagonistic activities mediated by competitive splicing mechanisms. In addition to alternative splicing, circRNAs expression may also be influenced by a complex network of regulatory processes, such as transcriptional modulation and degradation, which could potentially vary under different conditions^32^.

CircRNAs can function as scaffolds to regulate protein interactions^33^. Using bioinformatic analysis and RIP or RNA-pulldown assays, we determined that the BRPF3 protein was the binding target of circ-CELF1. Furthermore, we revealed that the PWWP domain of BRPF3(1075–1158aa) is crucial for its interaction with circ-CELF1.

The BRPF3 protein serves as a scaffold for the HATs complex and plays crucial roles in the regulation of gene transcription^25^. Nonetheless, it remains unclear whether BRPF3 influences the pathological progression of cardiac fibrosis. In the present study, we revealed that BRPF3 exerted a promotive effect on cardiac fibrosis. Furthermore, we examined the specific regulatory mechanism of circ-CELF1 on BRPF3 expression in CFs. The findings exhibited that knockdown of circ-CELF1 reduced the protein levels of BRPF3 rather than affecting the mRNA levels of BRPF3. The findings suggested that circ-CELF1 exerted its regulatory effect on the expression of BRPF3 primarily at the post-transcriptional level. The ubiquitin–proteasome system (UPS) is the key pathway for protein degradation in cells^34^. This study revealed that overexpression of circ-CELF1 inhibited the ubiquitination process of BRPF3, while the silencing of circ-CELF1 enhanced the ubiquitination of BRPF3 in response to treatment with a proteasome inhibitor (MG132). The above evidence substantiated the upregulation of BRPF3 protein expression through circ-CELF1-mediated modulation of the UPS. Additionally, BRPF3, a scaffold for the HATs complex, can regulate gene transcription in the nucleus^25^. The existing studies suggest that circRNAs possess the capability to induce modifications in protein subcellular localization^35^. Here, we discovered that overexpression of circ-CELF1 promoted the nucleus translocation of the BRPF3 protein. Besides, TGF-*β*1 promoted BRPF3 protein translocation into the CFs nucleus.

Histone acetylation is dynamically regulated by histone acetyltransferases (HATs) and deacetylases (HDACs)^36^. HATs are classified into families, such as p300/CBP, MYST, and GCN5, based on structural and sequence similarities^37^. KAT7, also known as HBO1 and MYST2, belongs to the MYST family^38^. The acetylation preference of KAT7 is directed towards lysine 5 and lysine 12 on histone H4, as well as lysine 14 on histone H3^29^. The findings of previous studies have demonstrated that BRPF3 plays a crucial role in regulating the stability of KAT7 protein by inhibiting Huwe1-mediated degradation in embryonic stem cells^26^. In our current study, we demonstrated that BRPF3 functioned as a modular scaffold to recruit histone acetyltransferase KAT7 and participated in chromatin remodeling, leading to an increase in H3K14 acetylation at target gene promoters. This unveils a novel mechanism underlying the role of BRPF3.

The RNA-binding protein CELF1 is crucial in various pathological conditions, including kidney and liver fibrosis and cardiac hypertrophy^39^, ^40^, ^41^. Notably, we found a significant positive correlation between circ-CELF1 expression and *Celf1* mRNA levels. Knockdown of circ-CELF1 resulted in the downregulation of *Celf1* mRNA expression. These findings prompted us to speculate on the existence of transcriptional regulation between KAT7 and CELF1, thereby establishing a feedback loop in circ-CELF1 production. Indeed, our study demonstrated that KAT7 enhances the transcription of *Celf1* by upregulating H3K14 acetylation levels.

However, the process of histone protein modifications may occur in a diverse array of genes under these conditions. H3K14ac histone modification may also directly regulate the gene transcription of fibrosis-related factors. Nonetheless, the precise mechanism by which fibrosis-related genes are regulated by H3K14ac histone modification remains unclear. More rigorous investigations, such as CHIP-seq or CUT&Tag analysis are required to address this question.

Emerging evidence has demonstrated that the expression of SMAD7, a negative regulator of TGF-*β*1 signaling, is increased in CELF1-silenced cells^30^. CELF1 serves as a crucial regulatory factor for profibrotic activation^39^. Thus, we investigated the impact of CELF1 on the expression of SMAD7 in CFs. Notably, our findings align with recent studies showing that CELF1 knockdown increases the mRNA and protein levels of SMAD7. Moreover, CELF1 further enhanced the transcription and back-splicing of *Celf1* pre-mRNA, thereby establishing a regulatory loop for circ-CELF1 production.

New findings suggest that circular RNA has a pivotal function in regulating the expression of host genes and forming feedback loop mechanisms, thereby contributing to the pathogenesis of diseases. For instance, circPVT1 facilitates nasopharyngeal carcinoma metastasis through the *β*-TrCP/c-Myc/SRSF1 positive feedback loop^42^. Additionally, the circ-ROBO1/KLF5/FUS feedback loop orchestrates liver metastasis in breast cancer^43^. Furthermore, a SERPINH1/c-Myc positive feedback loop expedites nasopharyngeal cancer carcinogenesis through splicing factor-derived circular RNA circ-CAMSAP1^44^. However, limited information has been reported about the underlying mechanisms of the circular RNAs feedback loop in cardiac fibrosis. Therefore, it is crucial to elucidate the existence of a feedback loop pathway that regulates the biogenesis of circRNAs in cardiac fibrosis. The findings of our study unveil a novel mechanism by which the CELF1/circ-CELF1/BRPF3/KAT7 feedback loop facilitates the progression of cardiac fibrosis.

Additionally, it is necessary to acknowledge the limitations of our study. While we successfully demonstrated the role of circ-CELF1 in mouse myocardial fibrosis using myocardial fibroblast-specific AAV9, we have not investigated the specific role of circ-CELF1 in myocardial fibroblasts knocking out transgenic mice, which represents a limitation of the present study. To demonstrate the efficiency of AAV9 with a periostin core promoter in fibroblasts, we isolated cardiac fibroblasts from adult mice and evaluated the physiological role of circ-CELF1 in cardiac fibroblasts. The findings of our study also demonstrate an elevated presence of circ-CELF1 in mouse plasma and human CFs, suggesting its potential as a diagnostic biomarker and therapeutic target for cardiac fibrosis. However, due to practical limitations, including ethical approval processes, difficulty in sample acquisition, and time constraints, we are currently unable to obtain the cardiac fibrosis samples we need. Nevertheless, with the continuous advancement of tissue engineering techniques, organoid models hold promise in predicting the diagnostic and therapeutic implications of circ-CELF1 in cardiac fibrosis.

## 5 Conclusions

Altogether, our findings demonstrate that the CELF1/circ-CELF1/BRPF3/KAT7 feedback loop is essential for cardiac fibrosis. We are the first to elucidate the crosstalk between a feedback loop and histone acetylation, providing a more comprehensive understanding of the mechanism underlying circ-CELF1 in cardiac fibrosis. These findings enhance our understanding of cardiac fibrosis progression and offer new perspectives on circRNA-based diagnostic and therapeutic strategies.

## Author contributions

Chaoqian Xu, Baofeng Yang and Mingyu Zhang: Conceptualization and Funding acquisition. Yuan Jiang, Bowen Zhang and Bo Zhang: Formal analysis, Writing - Original Draft, Writing - Review & Editing, Investigation and Methodology. Xiaoxiang Guan: Writing - Review & Editing and Supervision. Yuan Jiang, Xinhua Song, Wei Zeng, Liyang Zuo and Xinqi Liu: Visualization and Validation. Xiangyu Wang, Yang Qiao, Wenzheng Cheng, Saidi Jin, Dongni Ji, Zheng Dong, Xiaofei Guo, Xieyang Gong: Methodology. Lihua Sun, Lina Xuan, Rong Zhang and Berezhnova Tatjana Alexandrovna: Resources and Supervision.

## Conflicts of interest

The authors declare no conflicts of interest.

## Appendix A Supporting information

The following is the Supporting Information to this article:
