# Supplementary material for: A novel feedback loop: CELF1/circ-CELF1/BRPF3/KAT7 in cardiac fibrosis
Source: Acta Pharm Sin B. 2025 Jul 29;15(10):5192–211. doi: 10.1016/j.apsb.2025.07.036 (PMC12541625; doi:10.1016/j.apsb.2025.07.036)
Supplement: Multimedia component 1 [file mmc1.pdf]

Supporting Information for

Original article

A novel feedback loop: CELF1/circ-CELF1/BRPF3/KAT7 in cardiac fibrosis

Yuan Jiang[a,b,c,†], Bowen Zhang[a,b,d,†], Bo Zhang[a,b,†], Xinhua Song[a,b], Xiangyu Wang[a,b], Wei Zeng[a,b,c], Liyang Zuo[a,b], Xinqi Liu[a,b], Zheng Dong[a,b], Wenzheng Cheng[a,b], Yang Qiao[a,b], Saidi Jin[a,b], Dongni Ji[a,b], Xiaofei Guo[a,b,d], Rong Zhang[a,b], Xieyang Gong[e], Lihua Sun[a,b], Lina Xuan[a,b], Berezhnova Tatjana Alexandrovna[f], Xiaoxiang Guan[a,b,g,*], Mingyu Zhang[a,b,*], Baofeng Yang[a,b,*], Chaoqian Xu[a,b,*]

[a]*State Key Laboratory of Frigid Zone Cardiovascular Diseases (SKLFZCD), Department of Pharmacology (State Key Labratoray -Province Key Laboratories of Biomedicine-Pharmaceutics of China, Key Laboratory of Cardiovascular Research, Ministry of Education), College of Pharmacy, Harbin Medical University, Harbin 150081, China*

[b]*Department of Pharmacology, State-Province Key Laboratories of Biomedicine Pharmaceutics of China, Key Laboratory of Cardiovascular Medicine Research, Ministry of Education, College of Pharmacy, Harbin Medical University, Harbin 150081, China*

[c]*Department of Biopharmaceutical Sciences, College of Pharmacy, Harbin Medical University, Harbin 150081, China*

[d]*Institute of Clinical Pharmacy, the Second Affiliated Hospital, Harbin Medical University, Harbin 150081, China*

[e]*Department of Ultrasound, the Second Affiliated Hospital of Harbin Medical University, Harbin 150081, China*

[f]*Department of Pharmacology of Voronezh State Medical University, Voronezh 394018, Russia*

[g]*Institute of Clinical Pharmacy, the First Affiliated Hospital of Harbin Medical University, Harbin 150001, China*

Received 22 March 2025; received in revised form 20 April 2025; accepted 23 May 2025

*Corresponding authors.

E-mail addresses: xuchaoqian@ems.hrbmu.edu.cn (Chaoqian Xu), yangbf@ems.hrbmu.edu.cn (Baofeng Yang), zhangmingyu.302@163.com (Mingyu Zhang), guanxiaoxiang163@163.com (Xiaoxiang Guan).

[†]These authors made equal contributions to this work.

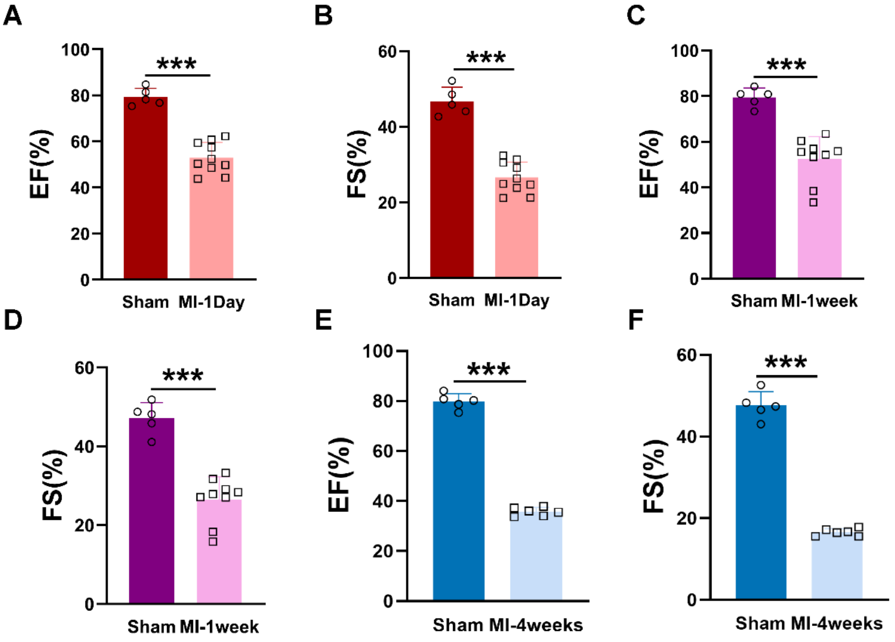

Figure S1. The echocardiography analysis was performed on Days 1,7 and 28 after MI surgery.

A–F. Echocardiographic analyses of the heart functions on Days 1, 7 and 28 after MI surgery, including ejection fraction (EF) and fraction shortening (FS) (n=5–10). Each data point in the figure represents a unique biological replicate. Statistical analysis was performed with Student's t test for two means. The data are presented as the mean ± SD. ***P<0.001.

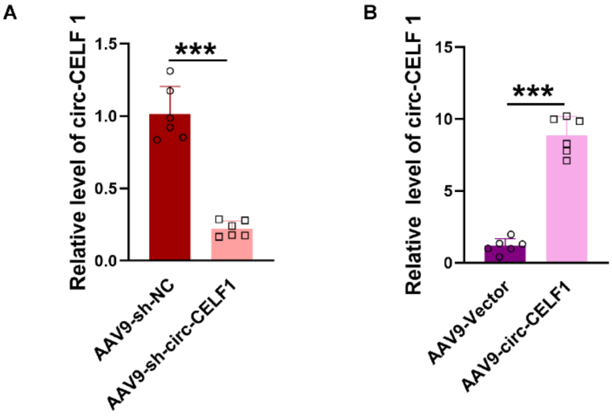

Figure S2. Verification of the effect of circ-CELF1 in mice.

A. Specific knockdown of circ-CELF1 by sh-circ-CELF1 in mouse heart tissues was verified (n=6). **B.** The mRNA level of circ-CELF1 overexpression in mouse heart tissue was verified using AAV9 viral particles carrying the full-length sequence of circ-CELF1 (n=6). Each data point in the figure represents a unique biological replicate. Statistical analysis was performed with Student's t test for two means. The data are presented as the mean ± SD. ***P<0.001.

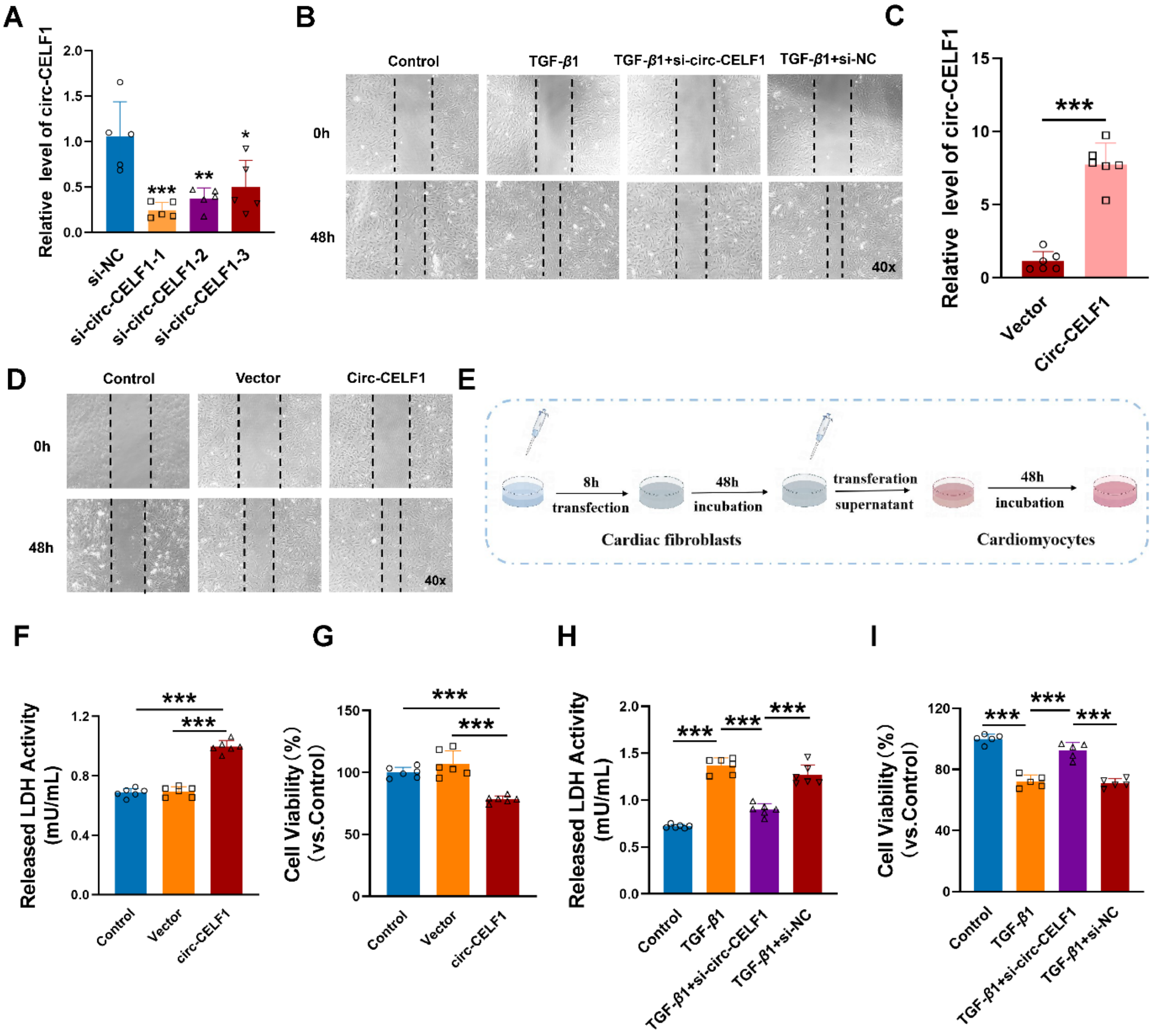

Figure S3. The intercellular communication between cardiomyocytes and cardiac fibroblasts.

A. Verification of the efficacy of circ-CELF1 in knocking down endogenous circ-CELF1 at the level in CFs (*n*=5). **B.** Wound healing assay was conducted to investigate the migration ability of CFs. Images were acquired at 0 and 48 h. **C.** Verification of circ-CELF1 overexpression at the level in CFs transfected with the overexpression plasmid (*n*=6). **D.** Wound healing assay was conducted to investigate the migration ability of CFs. Images were acquired at 0 and 48 h. **E.** The strategy to analyze the paracrine influence of CFs on cardiomyocytes. **F&H**. The LDH activity in cardiomyocytes was quantified using an LDH release assay kit (*n*=6). **G&I.** The viability of cardiomyocytes was assessed using the CCK-8 assay (*n*=6). Each data point in the figure represents a unique biological replicate. The data are presented as the mean ± SD. Statistical analysis was performed with one-way ANOVA followed by Bonferroni correction and Student's *t* test for two means. *P <0.05, **P<0.01, ***P<0.001.

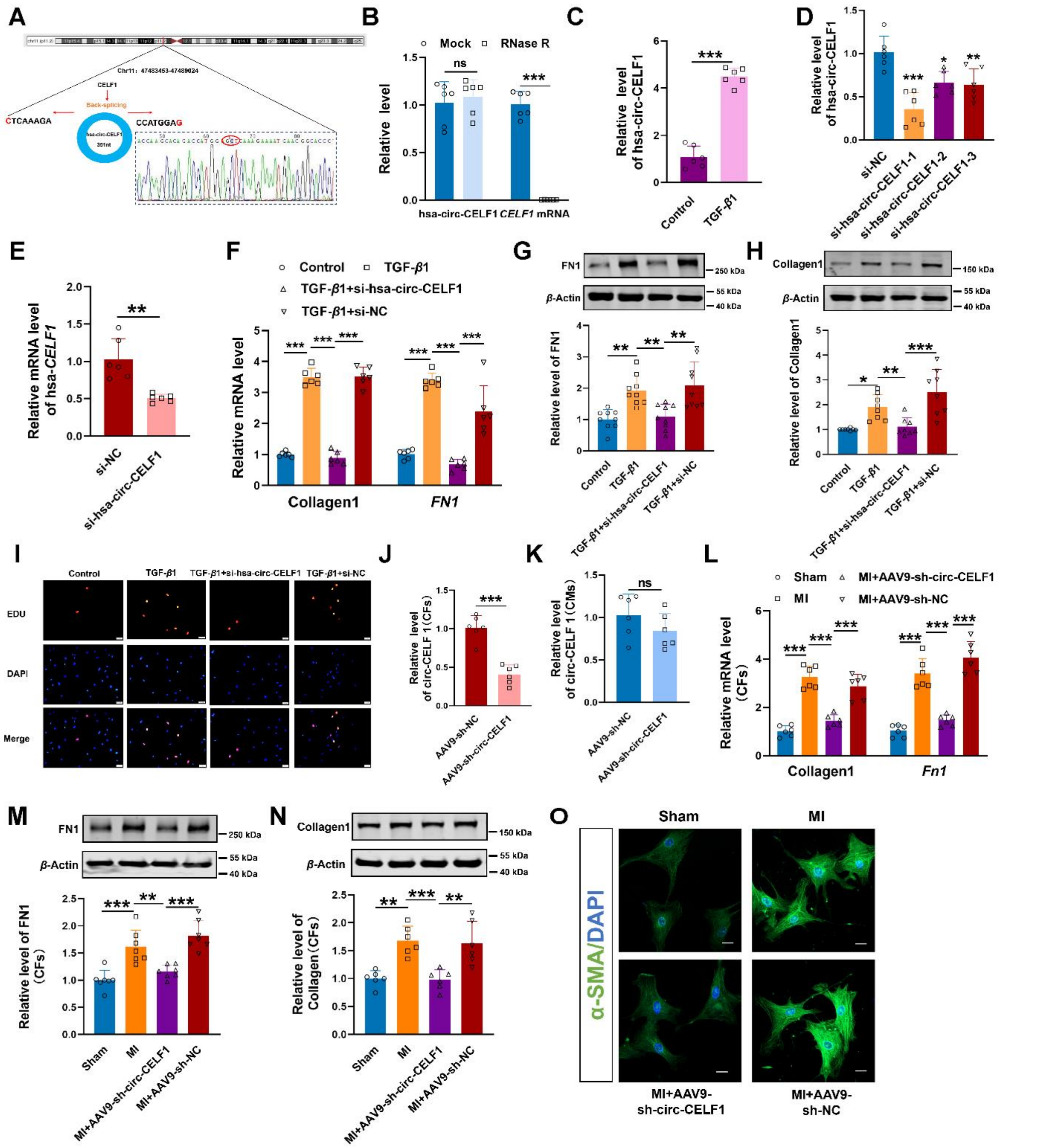

Figure S4. The pro-fibrotic effects of h-circ-CELF1 in HCFs and the physiological role of circ-CELF1 in isolated CFs.

A. The sanger sequencing is employed to identify the junction site of hsa-circ-CELF1. **B.** qPCR analysis of the level of hsa-circ-CELF1 and linear *CELF1* mRNA in HCFs following treatment with RNase R (*n*=6). **C.** The expression levels of hsa-circ-CELF1 in HCFs treated with TGF-*β*1 (*n*=6). **D.** Verification of the efficacy of hsa-circ-CELF1 in knocking down endogenous hsa-circ-CELF1 at the mRNA level in HCFs (*n*=6). **E.** qPCR assays of the mRNA expression of *hsa-CELF1* in hsa-circ-CELF1-silenced HCFs (*n*=6). **F.** qPCR assay for Collagen1 and *FN1* expression in HCFs. HCFs were transfected with hsa-circ-CELF1 siRNA or si-NC, and

followed by exposure to TGF-β1 for 24 h (n=6). **G–H.** Western blot analysis of the expression levels of Collagen1 and FN1 (n=8–9). **I.** Representative images of EdU staining in HCFs. The nuclei stained by DAPI (blue). Scale bar=100 μm. **J–K.** qPCR assays were conducted to detect the expression of circ-CELF1 in cardiomyocytes and fibroblasts isolated from adult mice (n=6). **L.** qPCR analysis of Collagen1 and *Fn1* expression in CFs isolated from adult mice (n=6). **M–N.** WB analysis of Collagen1 and FN1 expression levels in CFs isolated from adult mice (n=6–7). **O.** Immunofluorescence staining of α-SMA in CFs isolated from adult mice. Scale bar = 100 μm, α-SMA (green), nuclei (blue). Each datapoint in the figure represents a unique biological replicate. The data are presented as the mean ± SD. Statistical analysis was performed with one-way ANOVA followed by Bonferroni correction and Student's t test for two means. *P<0.05, **P<0.01, ***P<0.001.

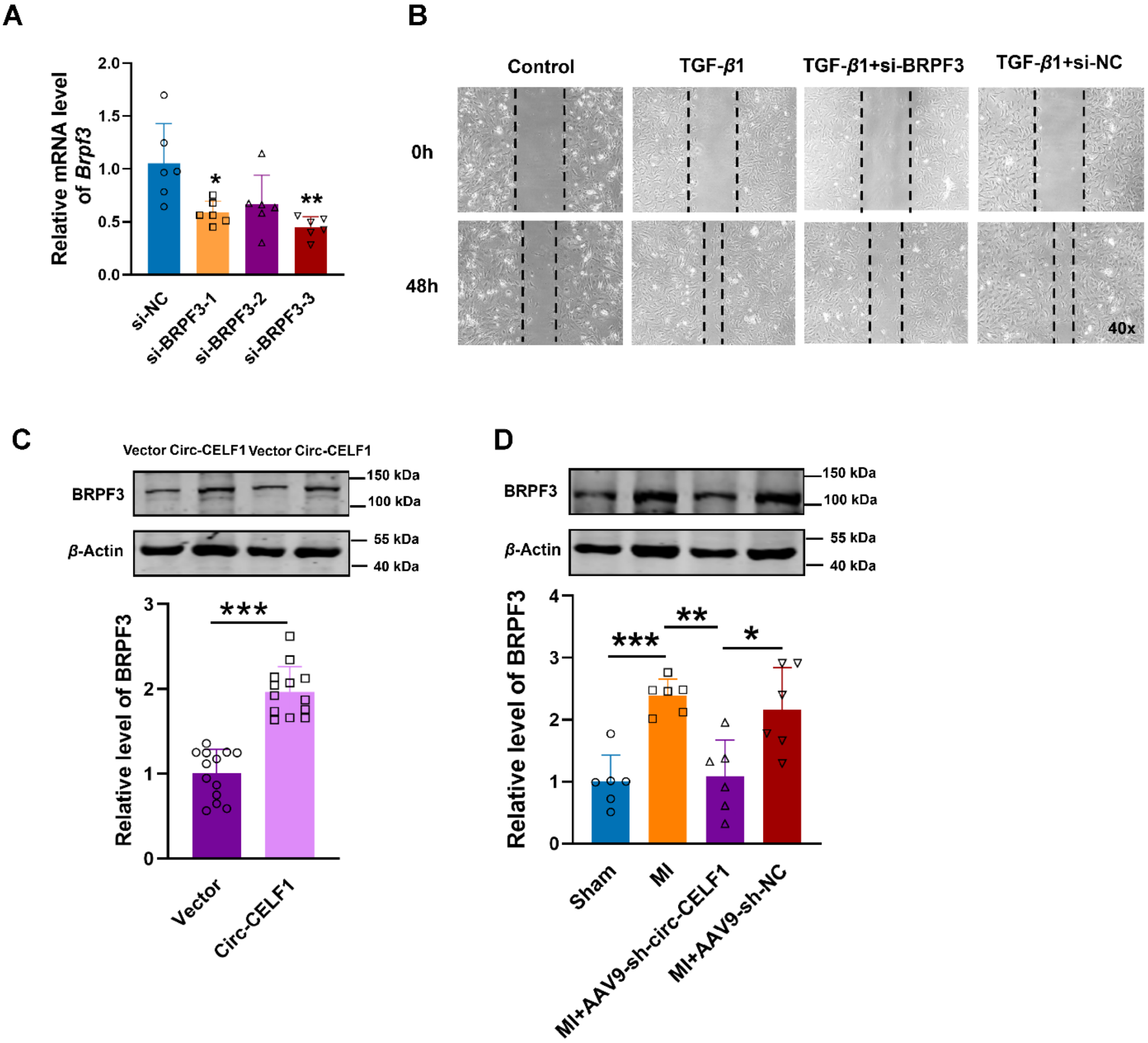

Figure S5. The expression levels of BRPF3 in mouse heart tissues and in CFs.

A. Verification of the efficacy in knocking down endogenous *Brpf3* at the mRNA levels in CFs relative to that of the scramble negative control (*n*=6). **B.** Wound healing assay was conducted to investigate the migration ability of CFs. Images were acquired at 0 and 48 h. **C.** WB analysis demonstrated the protein expression levels of BRPF3 in CFs overexpressing circ-CELF1 (*n*=13). **D.** WB assays detecting BRPF3 levels in mouse heart tissues after circ-CELF1 knockdown post MI surgery (*n*=6). Each data point in the figure represents a unique biological replicate. The data are presented as the mean ± SD. Statistical analysis was performed with one-way ANOVA followed by Bonferroni correction and Student's *t* test for two means. *$P<0.05$, **$P<0.01$, ***$P<0.001$.

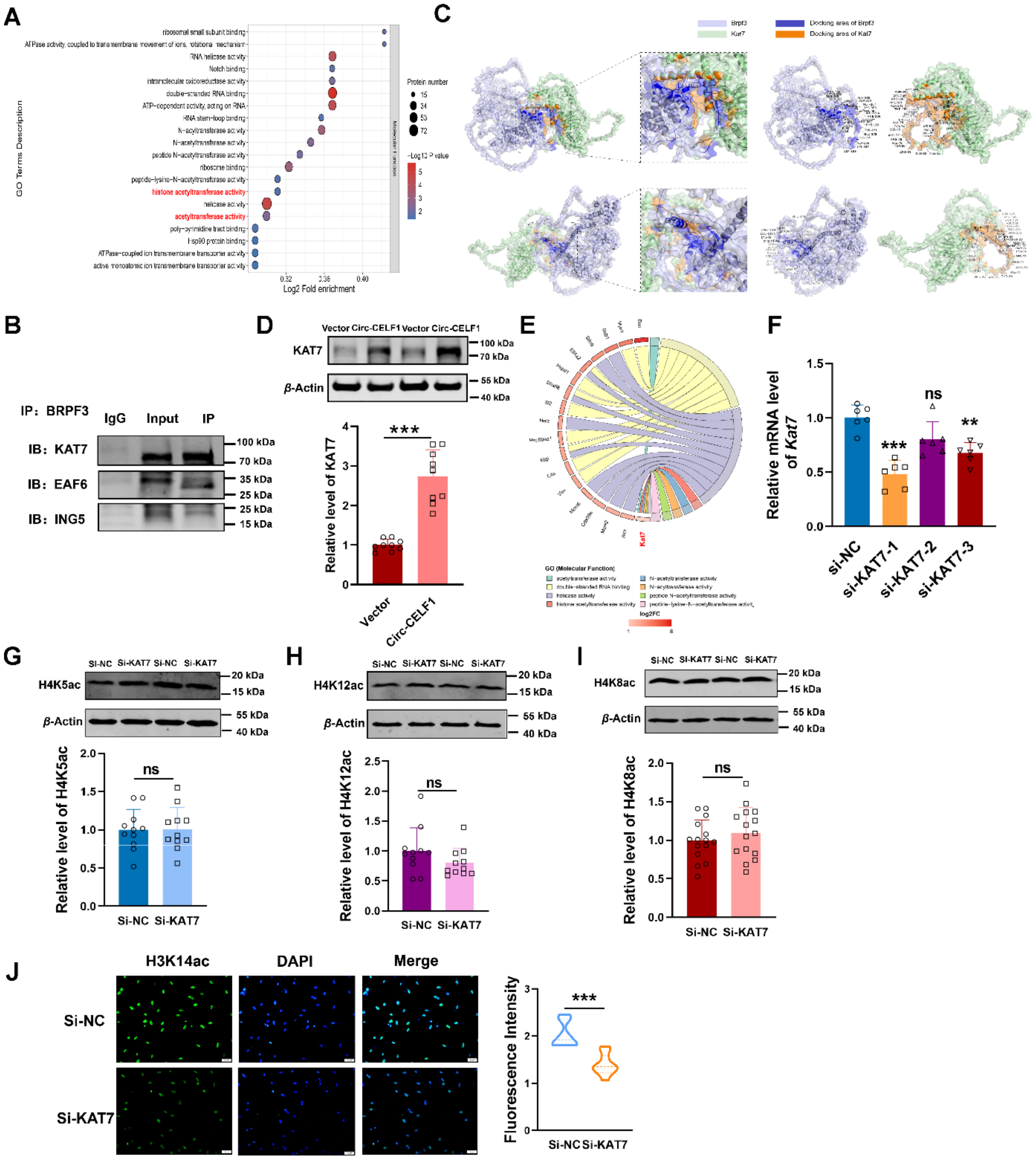

Figure S6. The acetylation status of H3 and H4 tails in CFs with KAT7 knockdown.

A. GO enrichment analysis of BRPF3-associated proteins. **B.** Co-IP and WB analysis for candidate proteins KAT7, EAF6, and ING5 in CFs. **C.** Analysis of the docking model for protein–protein interactions between BRPF3 and KAT7. **D.** WB analysis demonstrated the protein expression levels of KAT7 in CFs overexpressing circ-CELF1 (n=9). **E.** GO analysis revealed the functional enrichment associated with KAT7. **F.** Verification of the efficacy in knocking down endogenous *Kat7* at the mRNA levels in CFs relative to that of the scramble negative control (n=6). **G–I.** Western blot revealed H4K5ac, H4K12ac and H4K8ac expression in KAT7-silenced CFs (n=11–15). **J.** Immunofluorescence staining for H3K14ac (green) and

DAPI (blue) in KAT7-silenced CFs (n=10). Each data point in the figure represents a unique biological replicate. The data are presented as the means ± SD. Statistical analysis was performed with one-way ANOVA followed by Bonferroni correction and Student's t test for two means. **P<0.01, ***P<0.001.

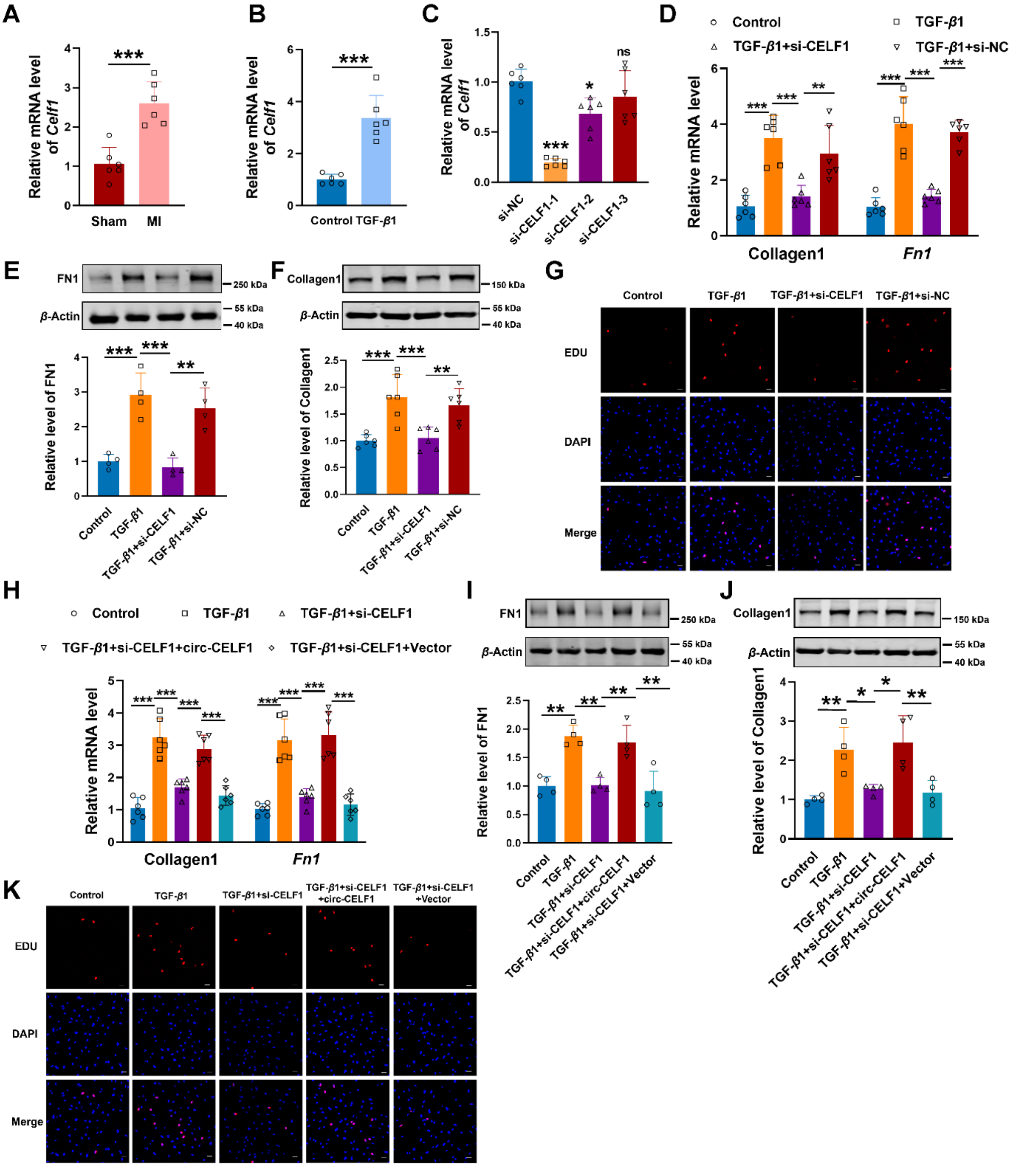

Figure S7. CELF1 modulates cardiac fibrosis via the circ-CELF1

A&B. qPCR analysis of *Celf1* mRNA expression in MI hearts and TGF-β1-treated CFs (n=6). **C.** Verification of the efficacy of *Celf1* in knocking down endogenous *Celf1* at the level in CFs (n=6). **D.** qPCR analysis of Collagen1 and *Fn1* expression in CFs transfected with *Celf1* siRNA

or si-NC and exposed to TGF-β1 for 24 h (n=6). **E&F.** WB analysis of Collagen1 and FN1 expression in CFs (n=4–6). **G.** Representative EdU staining images in CFs, with DAPI (blue) for nuclei. **H.** qPCR analysis was conducted to evaluate the expression levels of Collagen1 and *Fn1* in cardiac fibroblasts (CFs) transfected with *Celf1* siRNA or a circ-CELF1 overexpression plasmid, and subsequently exposed to TGF-β1 for 24 h (n=6). **I&J.** WB analysis of Collagen1 and FN1 expression in CFs (n=4). **K.** Representative EdU staining images in CFs, with DAPI (blue) for nuclei. Each data point in the figure represents a unique biological replicate. The data are presented as the mean ± SD. Statistical analysis was performed with one-way ANOVA followed by Bonferroni correction and Student's t test for two means. *P<0.05, **P<0.01, ***P<0.001.

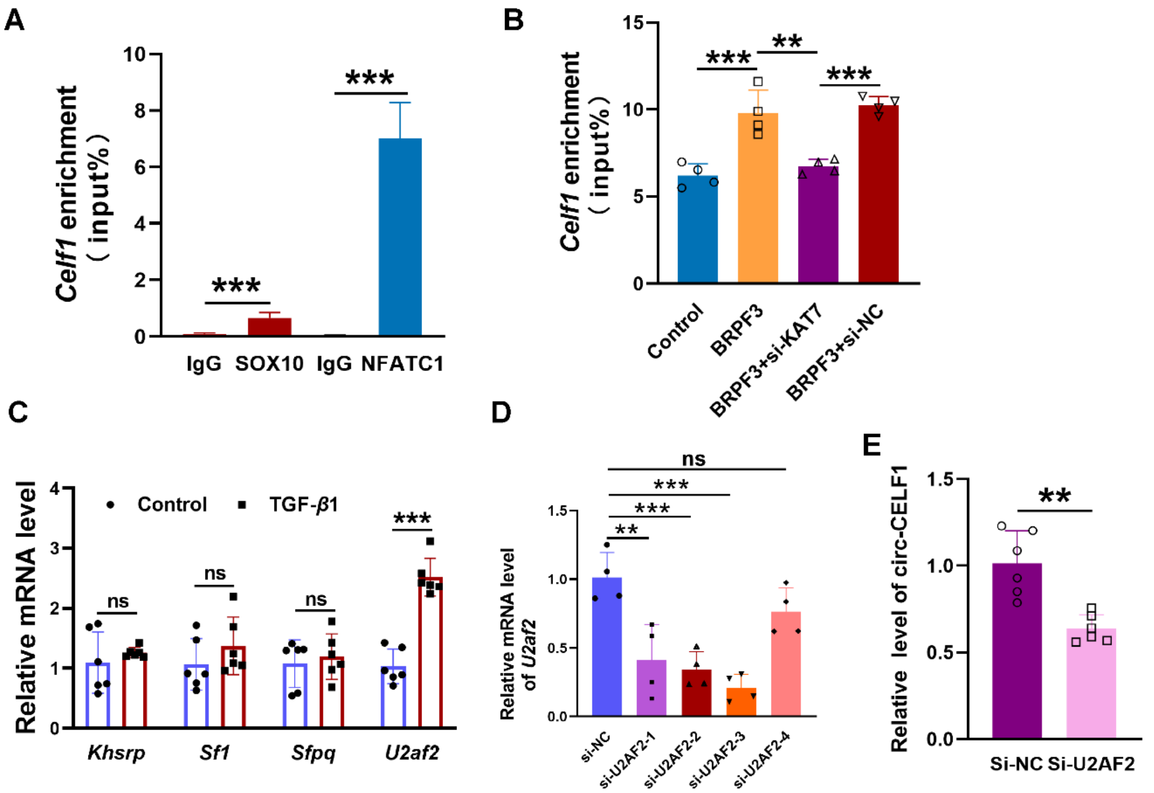

Figure S8. The transcription factors and splicing factors for CELF1

A. CHIP qPCR with SOX10 and NFATC1-specific antibodies to evaluate the enrichment of *Celf1* promoter regions in CFs (*n*=6). **B.** CHIP qPCR analysis demonstrating the enrichment of *Celf1* promoter regions by NFATC1-specific antibody in CFs co-transfected with the BRPF3 expression plasmid and KAT7 siRNA (*n*=4). **C.** The levels of four selected splicing factors, *Khsrp*, *Sf1*, *Sfpq and U2af2* in CFs treated with TGF-β1 (*n*=6). **D.** Verification of the efficacy in knocking down endogenous *U2af2* at the mRNA levels in CFs relative to that of the scramble negative control (*n*=4). **E.** qPCR revealed the circ-CELF1 expression in U2AF2-silenced CFs (*n*=6). Each data point in the figure represents a unique biological replicate. The data are presented as the means ± SD. Statistical analysis was performed with one-way ANOVA followed by Bonferroni correction and Student's *t* test for two means. **P<0.01, ***P<0.001.

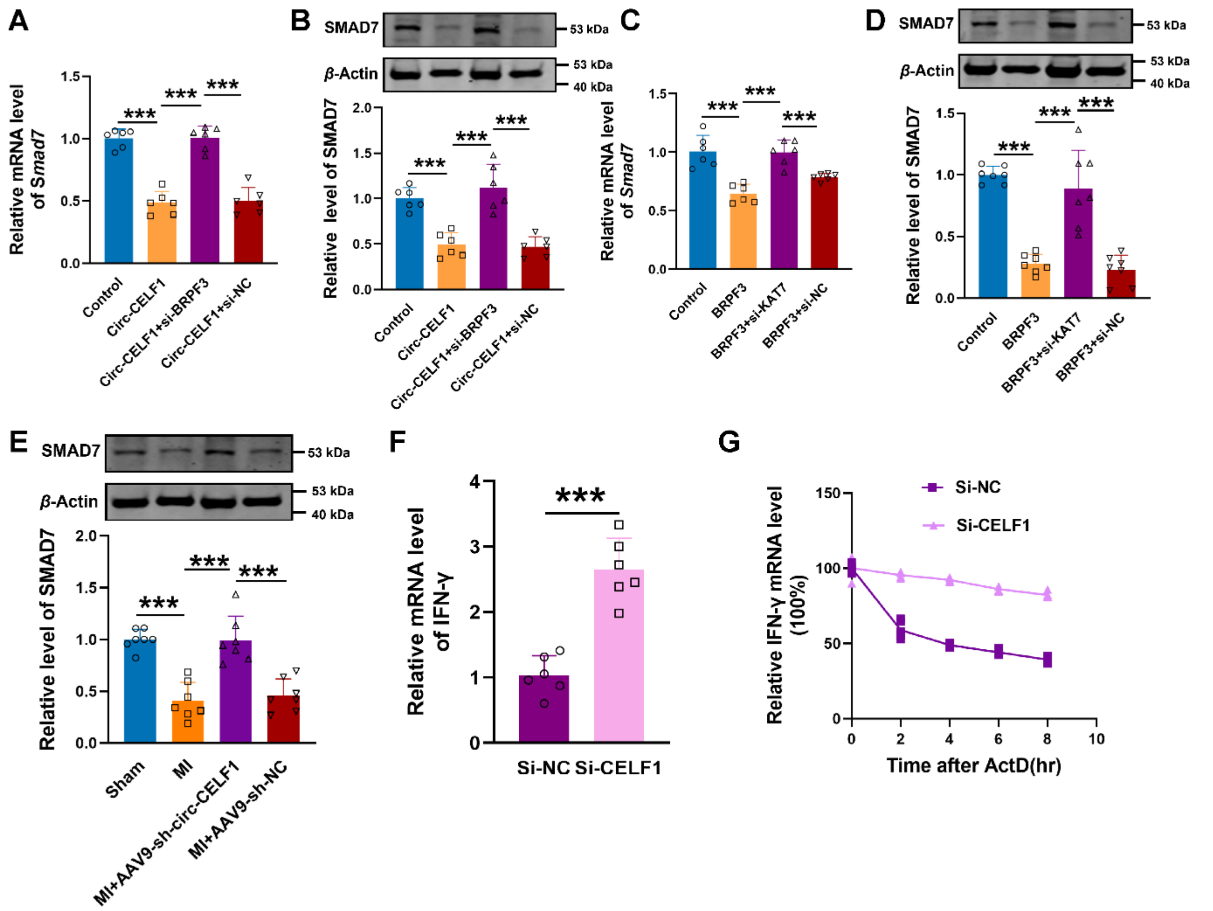

Figure S9. The effect of CELF1/circ-CELF1/BRPF3/KAT7 axis on SMAD7 expression in CFs.

A&B. qPCR and WB analysis of SMAD7 mRNA and protein levels in CFs transfected with circ-CELF1 and BRPF3 siRNA (n=6). **C&D.** qPCR and WB analysis of SMAD7 mRNA and protein levels in CFs transfected with BRPF3 and KAT7 siRNA (n=6–7). **E.** WB assays detecting SMAD7 levels in mouse heart tissues after circ-CELF1 knockdown post MI surgery (n=7). **F.** qPCR showed the mRNA level of IFN-γ in CELF1-silenced CFs (n=6). **G.** At the indicated time points (0, 2, 4, 6 and 8 h), the mRNA levels of IFN-γ with or without CELF1 depletion in the presence of the transcription inhibitor actinomycin D were quantified by qPCR (n=6). Each data point in the figure represents a unique biological replicate. The data are presented as the means ± SD. Statistical analysis was performed with one-way ANOVA followed by Bonferroni correction and Student's t test for two means. ***P<0.001.

Supporting Tables

Table S1 The following primers were used in this study.

Primer name	Primer sequence
circ-CELF1	Forward: AAGAACAGAAGAGAATGGCCCAG
	Reward: GCCGTTCATTTCTTTGAGTGC
Celf1	Forward: TCCAGCAGCAGTAATTCTGTCAACC
	Reward: ACCACCATTCAAAGCAGCCATCC
Collagen1	Forward: AAGAAGACATCCCTGAAGTCA
	Reward: TTGTGGCAGATACAGATCAAG
Fn1	Forward: AGTGGCTGAAGTCGCAAGGAAAC
	Reward: TAAGTCTGGGTCACGGCTGTCTC
Brpf3	Forward: CATCAAAGGGCAAGAGGAAGGAGTC
	Reward: ATCGGTAGTAAGCAGCAGGCAATG
Kat7	Forward: AGGACCTGATAGATGAGTGGATAGC
	Reward: TCGCTGGCTCGGAATGATAGG
β-Actin	Forward: CTACCTCATGAAGATCCTGACC
	Reward: CACAGCTTCTCTTTGATGTCAC
h-circ-CELF1	Forward: TCACAACATGAAAGTCCTCCCAG
	Reward: GTTCATTTTCTTTGAGCTCCATG
h-Collagen1	Forward: CAAGAACCCCAAGGACAAGA
	Reward: GTAGGTGATGTTCTGGGAGG
h-FN1	Forward: CACCACCAGCACCAGCACAC
	Reward: GGCTCATCTCCCTCCTCACTCAG
h-CELF1	Forward: GCCAACCTGTTCATCTACCACCTG
	Reward: GGCAGACACGACATTCCCAAAGG
h-β-Actin	Forward: CCTGGCACCCAGCACAAT
	Reward: GGGCCGGACTCGTCATAC
Khsrp	Forward: GACAAGCCACTGCGGATTATCG
	Reward: CACATCAATGCCTCCACCAACTC
Sf	Forward: AGAGGAGCCGATGGAACCAAG
	Reward: CGCAGTTTACGAGTCAGGTCTTC
Sfpq	Forward: GGTGGTGGTGGTGGCATAGG
	Reward: GCTCAGTACGCATGTCGCTTC
U2af2	Forward: CCGAGCACCATCAATCAGACAC
	Reward: CTCCTCATACTCCTCATCGTCCAG
IFN-γ	Forward: GCCAGATTATCTCTTTCTACCTCAGAC
	Reward: TGTGATTCAATGACGCTTATGTTGTTG
Smad7	Forward: GCAGGCTGTCCAGATGCTGT
	Reward: GATCCCCAGGCTCCAGAAGA

Table S2 Information on the antibodies used in this study.

Antibody	Dilution	Company
FN1	WB 1:500	Proteintech, USA
Collagen1	WB 1:500	Proteintech, USA
BRPF3	WB 1:500	Thermo Fisher Scientific, USA
	WB 1:1000	OriGene Technologies, USA
	IF 1:200	Bioss, China
UB	WB 1:500	Cell Signaling Technology, USA
Lamin B	WB 1:1000	Affinity Biosciences, USA
Tubulin	WB 1:1000	Proteintech, USA
KAT7	WB 1:500	Proteintech, USA
	IF 1:200	Proteintech, USA
EAF6	WB 1:500	Proteintech, USA
ING5	WB 1:500	Proteintech, USA
H3K14ac	WB 1:1000	PTOBIO, China
	IF 1:200	PTOBIO, China
H4K5ac	WB 1:1000	PTOBIO, China
H4K12ac	WB 1:1000	PTOBIO, China
H4K8ac	WB 1:1000	PTOBIO, China
SMAD7	WB 1:500	Proteintech, USA
β-Actin	WB 1:1000	Proteintech, USA
α-SMA	IF 1:400	Affinity Biosciences, USA
MMP2	WB 1:500	Proteintech, USA

Table S3 The sequences of small interfering RNAs.

siRNA name	siRNA sequence
si-Circ-CELF1-1 target	CCCAGTACTTAGCACTCAA
si-Circ-CELF1-2 target	CAGTACTTAGCACTCAAAG
si-Circ-CELF1-3 target	GTACTTAGCACTCAAAGAA
si-*Brpf3*-1 target	ACAACTACTGGCTGCTGAA
si-*Brpf3*-2 target	GAAGGAGGAGCTCAAATAC
si-*Brpf3*-3 target	GCACACATCTTTGCTGAAC
si-*Kat7*-1 target	ACCTGACAAGTGAATATGA
si-*Kat7*-2 target	ATGACAGAAGCGGACAATA
si-*Kat7*-3 target	CAAGGAGATCTCCATCAAA
si-*Celf1*-1 target	GGACAAAGAACAGAAGAGA
si-*Celf1*-2 target	GGCTTAAAGTGCAGCTCAA
si-*Celf1*-3 target	CCAGATCTTGATGCTATCA
si-h-Circ-CELF1-1	Sense: GCACAGACCAUGGAGCUCATT
	Antisense: UGAGCUCCAUGGUCUGUGCTT
si-h-Circ-CELF1-2	Sense: CAGACCAUGGAGCUCAAAGTT
	Antisense: CUUUGAGCUCCAUGGUCUGTT
si-h-Circ-CELF1-3	Sense: GACCAUGGAGCUCAAAGAATT
	Antisense: UUCUUUGAGCUCCAUGGUCTT
si-*U2af2*-1	Sense: GGCAGCUCAACGAGAAUAATT
	Antisense: UUAUUCUCGUUGAGCUGCCTT
si-*U2af2*-2	Sense: GUGAGUACGUGGACAUCAATT
	Antisense: UUGAUGUCCACGUACUCACTT
si-*U2af2*-3	Sense: CACGGUGGAUUGAUUCGUUTT
	Antisense: AACGAAUCAAUCCACCGUGTT
si-*U2af2*-4	Sense: GAAGGCCUCAUGACUAUCATT
	Antisense: UGAUAGUCAUGAGGCCUUCTT
